# CLCuMuB βC1 Subverts Ubiquitination by Interacting with NbSKP1s to Enhance Geminivirus Infection in *Nicotiana benthamiana*

**DOI:** 10.1371/journal.ppat.1005668

**Published:** 2016-06-17

**Authors:** Qi Jia, Na Liu, Ke Xie, Yanwan Dai, Shaojie Han, Xijuan Zhao, Lichao Qian, Yunjing Wang, Jinping Zhao, Rena Gorovits, Daoxin Xie, Yiguo Hong, Yule Liu

**Affiliations:** 1 MOE Key Laboratory of Bioinformatics, Center for Plant Biology, School of Life Sciences, Tsinghua University, Beijing, China; 2 College of Biological Sciences, China Agricultural University, Beijing, China; 3 Institute of Virology and Biotechnology, Zhejiang Academy of Agricultural Sciences, Hangzhou, China; 4 Institute of Plant Sciences and Genetics in Agriculture, Robert H. Smith Faculty of Agriculture, Food and Environment, Hebrew University of Jerusalem, Rehovot, Israel; 5 Research Centre for Plant RNA Signaling, College of Life and Environmental Sciences, Hangzhou Normal University, Hangzhou, China; Agriculture and Agri-Food Canada, CANADA

## Abstract

Viruses interfere with and usurp host machinery and circumvent defense responses to create a suitable cellular environment for successful infection. This is usually achieved through interactions between viral proteins and host factors. Geminiviruses are a group of plant-infecting DNA viruses, of which some contain a betasatellite, known as DNAβ. Here, we report that *Cotton leaf curl Multan virus* (CLCuMuV) uses its sole satellite-encoded protein βC1 to regulate the plant ubiquitination pathway for effective infection. We found that *CLCuMu betasatellite* (CLCuMuB) βC1 interacts with NbSKP1, and interrupts the interaction of NbSKP1s with NbCUL1. Silencing of either *NbSKP1s* or *NbCUL1* enhances the accumulation of CLCuMuV genomic DNA and results in severe disease symptoms in plants. βC1 impairs the integrity of SCF^COI1^ and the stabilization of GAI, a substrate of the SCF^SYL1^ to hinder responses to jasmonates (JA) and gibberellins (GA). Moreover, JA treatment reduces viral accumulation and symptoms. These results suggest that CLCuMuB βC1 inhibits the ubiquitination function of SCF E3 ligases through interacting with NbSKP1s to enhance CLCuMuV infection and symptom induction in plants.

## Author Summary

Viruses pose a serious threat to field crops worldwide; therefore, understanding the mechanisms of viral disease can help crop improvements. Here, we investigate how *Cotton leaf curl Multan virus* (CLCuMuV) interacts with plant to cause viral disease. We found that CLCuMuV uses its sole satellite-encoded protein βC1 to regulate the plant ubiquitination pathway for effective infection. By interrupting the interaction of NbSKP1 with NbCUL1 through its interaction of SKP1, βC1 interferes with the plant ubiquitination pathway and impairs plant hormone signallings to enhance viral accumulation and symptoms. These new insight into the mechanisms of viral disease may help crop improvements in the future.

## Introduction

Monopartite begomoviruses often possess an essential disease-specific betasatellite and are responsible for devastating diseases in many crops [1]. For example, at least six distinct begomoviruses that are associated with a single betasatellite, *Cotton leaf curl Multan betasatellite* (CLCuMuB), cause Cotton leaf curl disease (CLCuD), which is a major constraint to cotton production in Asia [2]. *Cotton leaf curl Multan virus* (CLCuMuV) is one of these begomoviruses and can infect cotton and many other plants including *Nicotiana benthamiana*. CLCuMuV consists of a circular single-stranded DNA genome that encodes only 6 proteins (V1 and V2 in virion-sense strand whilst C1, C2, C3 and C4 in virion complementary-sense strand). CLCuMuB is a small circular single-stranded DNA molecule that is essential for CLCuMuV to induce disease symptoms in plants [3].

Betasatellites, such as CLCuMuB, are approximately half the size of the begomovirus DNA genomes. They require the helper begomoviruses for replication and movement in plants and only encode a single multifunctional pathogenicity protein βC1 [1]. βC1 can up-regulate the proliferation of its cognate helper virus [4], and complement the movement function encoded by the DNA B component of some bipartite begomoviruses [5]. βC1 is essential for producing viral disease symptoms [4, 6–12] and plays important roles in suppression of transcriptional (TGS) [13] and posttranscriptional gene silencing (PTGS) [14–18]. Furthermore, βC1 can also promote the performance of the whitefly and impair plant development [19–22]. More details about the multiple functions of βC1 can be found in recently published reviews [1, 23]. However, how geminiviruses exploit βC1 to perform these diverse functions needs further investigations.

Ubiquitination is a highly dynamic posttranslational modification process that is a major protein degradation and rapid regulatory mechanism in plants [24]. Through the action of a sequential cascade of three enzymes consisting of E1, E2, and E3, ubiquitin is covalently attached to substrate proteins, and then, in most cases, the polyubiquitinated proteins will be degraded by the 26S proteosome. As the most abundant member of the E3 family, the SKP1/CUL1/F-box (SCF) complex is the best characterized multi-subunit ubiquitin ligase. In the SCF complex, SKP1/ASK1 (S-phase kinase-associated protein) acts as a bridge between CUL1 (Cullin1) and F-box proteins. CUL1 is the major structural scaffold and F-box proteins are responsible for recognizing target substrates. RBX1 is the fourth subunit that is heterodimerized with CUL1, and binds E2 through its RING Finger domain. More than 700 predicted F-box proteins are encoded by the *Arabidopsis thaliana* genome, suggesting these F-box proteins have highly targeting potentials for extensive regulatory functions [25, 26].

The SCF complex-based E3 ubiquitin ligases have been known to regulate plant hormone signaling. Several phytohormone receptors are F-box proteins in SCF complexes, such as SCF^TIR1^ for auxin, SCF^COI1^ for jasmonates, SCF^SLY1/GID2^ for gibberellins and SCF^MAX2^ for strigolactones [27–30]. In addition, SCF complexes regulate ethylene (ET) signal transduction at multiple points (SCF^ETP1^ and SCF^ETP2^ for EIN2, SCF^EBF1^ and SCF^EBF2^ for EIN3) [31, 32]. Since phytohormones have pivot functions in vegetative growth, compromising of these pathways is usually accompanied by abnormal developmental phenotype. Among them, JA plays a crucial role in defense against pathogens and insects. Recently, JA pathway was reported to be involved in plant defense against geminivirus infection [33].

In this study, we report that a geminivirus uses its satellite-encoded βC1 to interfere with the ubiquitination function of SCF E3 ligases to enhance viral infection and symptom development in plants.

## Results

### CLCuMuB βC1 Is Required for Development of Typical Disease Symptoms and Enhancement of CLCuMuV DNA Accumulation

CLCuMuB was reported to enhance DNA accumulation of the helper virus and be necessary for producing viral disease symptoms [4]. To see whether βC1 is responsible for these functions, we constructed a null mutant betasatellite for the βC1 gene [34] with a ATG-TGA transition in the start codon, hereafter called βM1 (S1 Fig). Different from *N*. *benthamiana* plants infected with CLCuMuV and β (CA+β) causing severe downward leaf curling and darkening as well as swollen veins, plants infected with CLCuMuV and βM1 (CA+βM1) grew taller, developed much milder symptoms and accumulated much less CLCuMuV genomic DNA (S2A and S2B Fig).

Further, we generated transgenic *N*. *benthamiana* plants expressing non-tagged or tagged βC1. However, most transgenic plants have very severe symptoms and are infertile or dead finally. Nevertheless, we were able to obtain five lines expressing non-tagged βC1 under control of its native promoter (*βC1pro*:*βC1*), 2 lines expressing GFP-tagged βC1 driven by CaMV 35S promoter (*35Spro*:*GFP-βC1*) and 4 lines expressing HA-tagged βC1 driven by CaMV 35S promoter (*35Spro*:*HA-βC1*). All these transgenic plants showed aberrant development phenotype (S3 Fig).

Taken together, these results suggest that CLCuMuB βC1 is required for development of typical disease symptoms and enhancement of CLCuMuV DNA accumulation.

### NbSKP1s Interacts with CLCuMuB βC1 *In Vitro* and *In Vivo*

**Fig 1:**
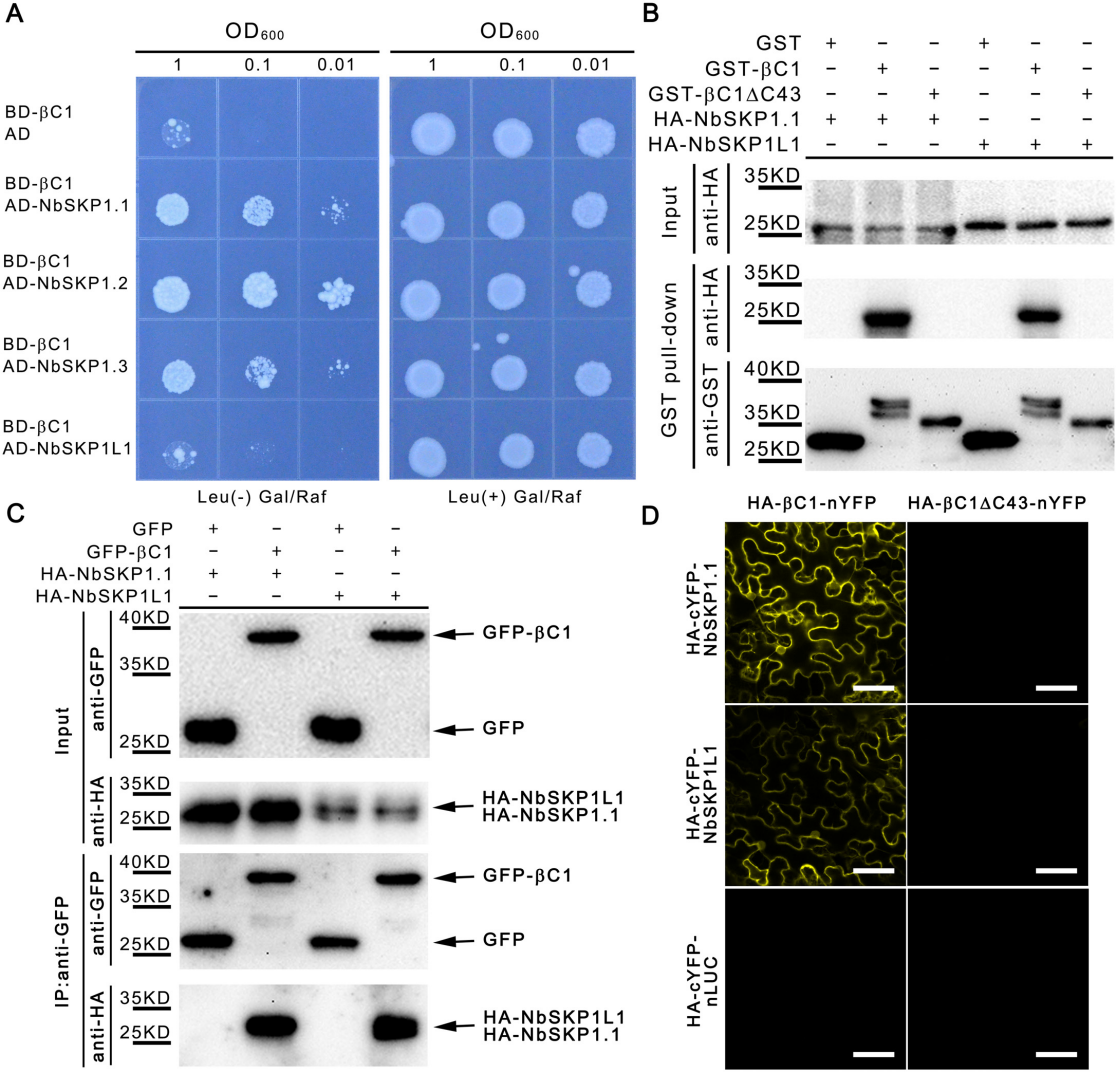
NbSKP1s interacts with CLCuMuB βC1 *in vitro* and *in vivo*. (A) Growth of SKY48 yeast strains containing NLS-LexA BD-CLCuMuB βC1 transformed with AD-NbSKP1s, AD-NbSKP1L1 or AD (control) on Leu-containing (Leu^+^) and Leu-deficient (Leu^−^) medium with galactose (Gal) and raffinose (Raf) at 28°C for 4 d. Yeast cells were plated at OD_600_ = 1, 0.1, 0.01. (B) *In vitro* GST pull-down assays. His-HA-NbSKP1.1 and His-HA-NbSKP1L1 were pulled down by GST-CLCuMuB βC1 (GST-βC1), GST or GST-βC1ΔC43. βC1ΔC43 represents a βC1 mutation with the deletion of C-terminal 43 amino acids. GST beads were washed and proteins were analyzed via SDS-PAGE and western blot assays using anti-GST and anti-HA antibodies. (C) Co-immunoprecipitation (co-IP) assays show that CLCuMuB βC1 interacted with NbSKP1.1 and NbSKP1L1 *in vivo*. GFP-tagged CLCuMuB βC1 (GFP-βC1) was co-expressed with 2×HA-tagged NbSKP1.1 or NbSKP1L1 (HA-NbSKP1.1 or HA-NbSKP1L1) in *N*.*benthamiana* leaves by agroinfiltration. GFP co-expressed with HA-NbSKP1.1 or HA-NbSKP1L1 was introduced as a negative control. At 48 hpi, leaf lysates were immunoprecipitated (IP) with GFP-Trap agarose, then the immunopercipitates were detected by western blotting using anti-GFP and anti-HA antibodies. (D) A confocal image of BiFC shows a positive result in leaf epidermal cells. NbSKP1.1 or NbSKP1L1 fused with HA and the C-terminal fragment of YFP (HA-cYFP-NbSKP1.1 or HA-cYFP-NbSKP1L1) was transiently co-expressed in leaves of *N*. *benthamiana* with CLCuMuB βC1 or βC1ΔC43 fused with HA and N-terminal fragment of YFP (HA-βC1-nYFP or HA-βC1ΔC43-nYFP). Bar scale represents 40 μm. Photos were imaged at 48 hpi using a Zeiss LSM 710 laser scanning microscope. nLUC represents the N-terminal fragment of firefly luciferase.

To understand how CLCuMuB βC1 facilitates virus infection, we used CLCuMuB βC1 as bait in a yeast two-hybrid (Y2H) system [35] to identify host CLCuMuB βC1-interacting proteins. From screening the *Solanum lycopersicum* cDNA library, we characterized a full-length SKP1-like protein (designated as *SlSKP1*) that interacted with βC1. Furthermore, 12 putative *NbSKP1* homologues identified in the *N*. *benthamiana* genome through bioinformatics analysis (http://solgenomics.net), encode proteins with more than 44% amino-acid identity to SlSKP1. However, we obtained only 4 predicted cDNAs by RT-PCR. Indeed, RNA-seq results (ftp://ftp.solgenomics.net/transcript_sequences/by_species/Nicotiana_benthamiana/) indicates that other 8 putative homologues are not or rarely expressed in leaf tissues. Three of the 4 NbSKP1 homologues NbSKP1.1, NbSKP1.2 and NbSKP1.3, collectively called NbSKP1s, interact with CLCuMuB βC1, whilst the other do not or interact very weakly with βC1 in yeasts and it is named as NbSKP1L1 (NbSKP1-like 1) (Fig 1A). NbSKP1.1 shares 95.5%, 91.7% and 44.9% amino-acid identity to NbSKP1.2, NbSKP1.3 and NbSKP1L1, respectively (S4 Fig).

To examine whether CLCuMuB βC1 directly interacts with NbSKP1.1, *in vitro* GST pull-down assay was performed. His-HA double-tagged NbSKP1.1 (His-HA-NbSKP1.1) was expressed in *E*. *coli* BL21 (DE3) and then purified by Ni-NTA Agarose (Qiagen, Netherlands) column. After elution, His-HA-NbSKP1.1 was incubated with Glutathione Sepharose 4B (GE, American) bonded with *E*. *coli-*expressed GST, GST-tagged CLCuMuB βC1 (GST-βC1) or its mutant with the deletion of C-terminal 43 amino acids (GST-βC1ΔC43). His-HA-NbSKP1.1 was pulled down by GST-βC1 but not GST and GST-βC1ΔC43 (Fig 1B), indicating that NbSKP1.1 can directly interact with βC1. To our surprise, His-HA double-tagged NbSKP1L1 (His-HA-NbSKP1L1) was also pulled down by GST-βC1 but not GST and GST-βC1ΔC43. (Fig 1B).

We also demonstrated *in planta* interaction of CLCuMuB βC1 with NbSKP1.1 using co-immunoprecipitation (Co-IP) assay. In this assay, HA-tagged NbSKP1.1 (HA-NbSKP1.1) was co-expressed transiently with GFP or GFP-tagged CLCuMuB βC1 (GFP-βC1) in *N*. *benthamiana* by agroinfiltration. GFP-βC1 transgenic *N*.*benthamiana* exhibits leaf curl symptoms, which indicates GFP-βC1 is a functional protein (S3C Fig). Total protein extracts were immunoprecipitated by GFP-Trap beads (ChromoTek, German). The resulting precipitates were analyzed by western blot assays using an anti-HA antibody (CST, USA). We found that HA-NbSKP1.1 was co-immunoprecipitated by GFP-βC1 but not GFP (Fig 1C). Similarly, we also found that HA-tagged NbSKP1L1 (HA-NbSKP1L1) was co-immunoprecipitated by GFP-βC1 but not GFP (Fig 1C). To confirm these Co-IP results, we performed the reverse IP. GFP-βC1 was co-expressed transiently with HA-tagged GUS (HA-GUS), HA-NbSKP1.1 or HA-NbSKP1L1 in *N*. *benthamiana* by agroinfiltration. Total protein extracts were immunoprecipitated by HA-beads (Abmart, China). The resulting precipitates were analyzed by western blot assays using an anti-GFP antibody (ChromoTek, German). GFP-βC1 was pulled down by HA-NbSKP1.1 and HA-NbSKP1L1 but not HA-GUS (S5A Fig).

To find where CLCuMuB βC1 interacts with NbSKP1.1 and NbSKP1L1 in plant cells, citrine yellow fluorescent protein (YFP)-based bimolecular fluorescence complementation (BiFC) assays [36] were performed. HA-tagged βC1 or βC1ΔC43 was fused to the N-terminal domain of YFP (nYFP) to generate HA-βC1-nYFP or HA-βC1ΔC43-nYFP. NbSKP1.1, NbSKP1L1 and the N-terminal fragment of firefly luciferase (nLUC) as a negative control were fused to HA-tagged C-terminal domain of YFP (HA-cYFP) to generate HA-cYFP-NbSKP1.1, HA-cYFP-NbSKP1L1 and HA-cYFP-nLUC. Western blot assays using an anti-HA antibody showed that all chimeric proteins can be expressed correctly (S5B Fig). HA-βC1-nYFP or HA-βC1ΔC43-nYFP was transiently co-expressed with HA-cYFP-NbSKP1.1, HA-cYFP-NbSKP1L1 or HA-cYFP-nLUC respectively in *N*. *benthamiana*. No such interaction between HA-βC1-nYFP and HA-cYFP-nLUC was found. However, positive interactions between HA-βC1-nYFP and HA-cYFP-NbSKP1.1 or HA-cYFP-NbSKP1L1 were observed in both nucleus and cell periphery, as indicated by occurrence of yellow fluorescence (Fig 1D). As a control, HA-βC1ΔC43-nYFP didn’t interact with HA-cYFP-NbSKP1.1 or HA-cYFP-NbSKP1L1 (Fig 1D).

Taken together, these results demonstrate that NbSKP1s and NbSKP1L1 interact with CLCuMuB βC1 both *in vitro* and *in vivo*, and the interaction of the two proteins occurs in nucleus and cell periphery of plant cells.

### The N-terminal Domain of NbSKP1.1 Is Responsible for the Interaction with CLCuMuB βC1

**Fig 2:**
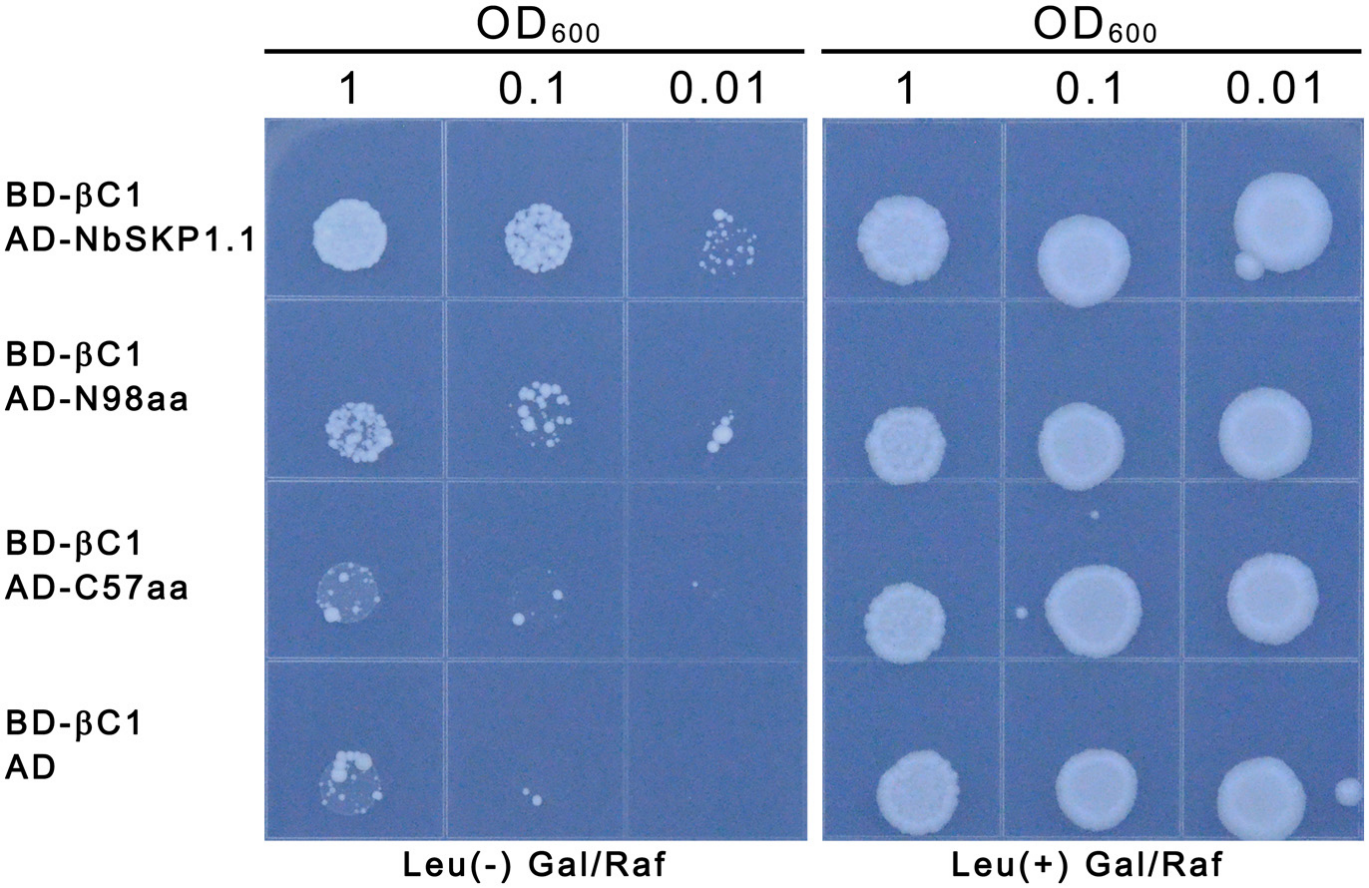
The N-terminal domain of NbSKP1.1 interacts with CLCuMuB βC1 in yeast. Growth of SKY48 yeast strains containing NLS-LexA BD-CLCuMuB βC1 (BD-βC1) transformed with AD fused full length, N-terminal fragment (N98aa), C-terminal fragment (C57aa) of NbSKP1.1 or AD (control) on Leu-containing (Leu^+^) and Leu-deficient (Leu^−^) medium with galactose (Gal) and raffinose (Raf) at 28°C for 6 d. Yeast cells were plated at OD_600_ = 1, 0.1, 0.01.

The crystal structures of human SKP1 [37] and *Arabidopsis* ASK1 [38] suggest that SKP1 can be divided into N-terminal and C-terminal domains. The N-terminal BTB-POZ domain of SKP1 is responsible for its binding to CUL1 whilst its C-terminal domain is thought to be essential for SKP1 to interact with F-box proteins. The Y2H assays showed that CLCuMuB βC1 interacted with the first 98 amino-acid N-terminal region of NbSKP1.1 (N98aa), but not with the C-terminal region (aa 99–155) of NbSKP1.1(C57aa), as indicated by growth of yeast on Leu^−^ plates containing galactose (Gal) and raffinose (Raf) (Fig 2).

### CLCuMuB βC1 Interferes with the Interaction between NbSKP1.1 and NbCUL1

**Fig 3:**
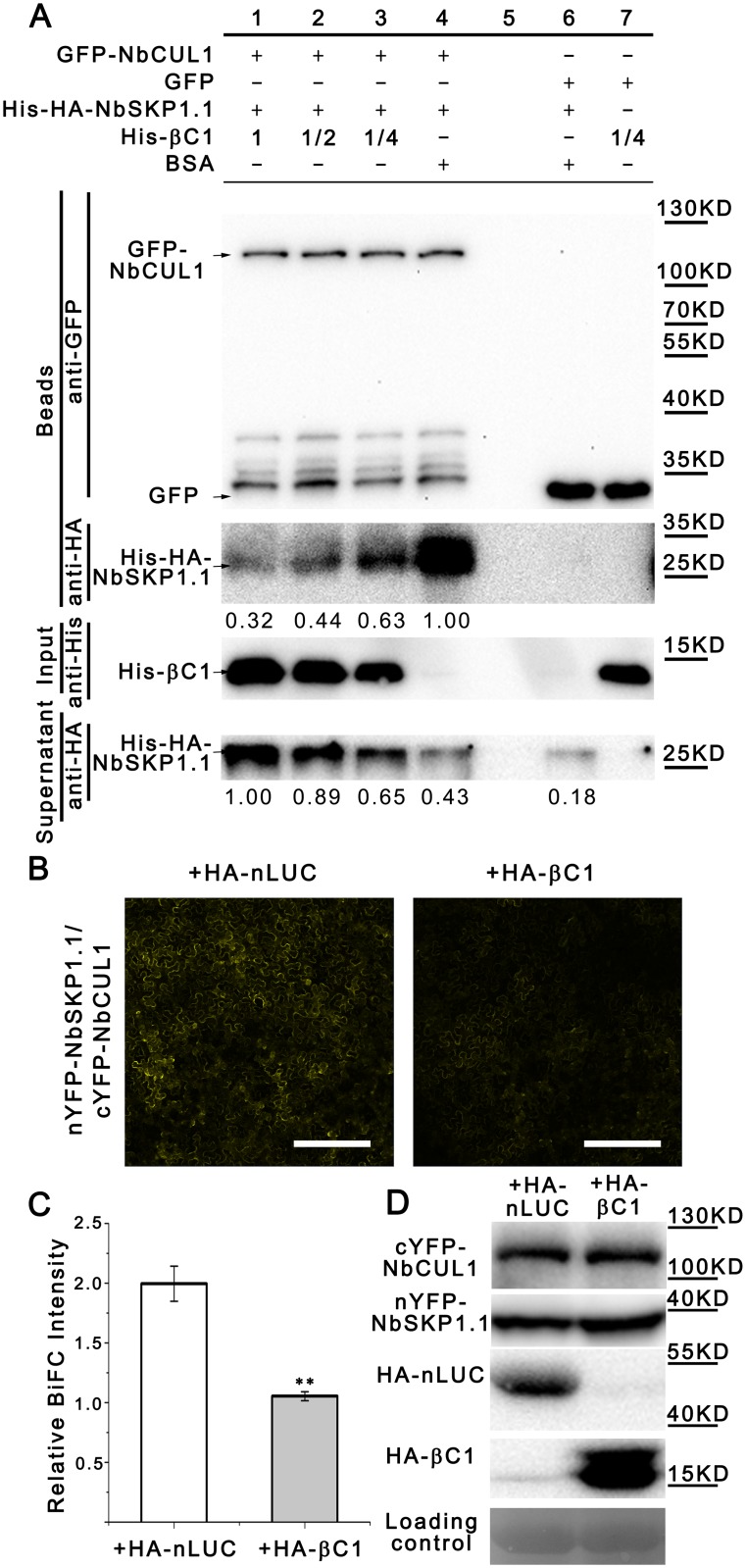
CLCuMuB βC1 interferes with the interaction between NbCUL1 and NbSKP1.1 *in vitro* and *in vivo*. (A) GFP competitive pull-down assay *in vitro*. His-βC1 was expressed in *E*. *coli* as inclusion body and refolded through urea-arginine dialysis. BSA (NEB, USA) was used as a control. GFP-NbCUL1 or GFP was expressed in *N*. *benthamiana* leaves and trapped through GFP-Trap agarose. After the supernatant was discarded, GFP-Trap agarose was incubated with *E*. *coli*-expressed His-HA-NbSKP1.1, then the supernatant was discarded. GFP-Trap agarose was incubated with gradient dilutions (1, 1/2, 1/4) of His-βC1. Finally, agarose was washed and proteins were analyzed via SDS-PAGE and western blot assays using anti-GFP and anti-HA antibodies. Input was analyzed by the anti-His antibody (EASYBIO, China) and supernatant was analyzed by the anti-HA antibody. Intensity was detected through Total Lab TL120. (B) A confocal image of BiFC assays show that CLCuMuB βC1 interfered with the interaction between NbCUL1 and NbSKP1.1 *in vivo*. Photos were taken at 48 hpi. Bar scale represents 200 μm. (C) BiFC intensity (means±SEM, n = 4) was quantified by YFP fluorescence. Relative BiFC intensity was normalized to the control. The raw data were analyzed by two-sample *t*-test to show the significance level at 0.01 (**). (D) The protein level of cYFP-NbCUL1 and nYFP-NbSKP1.1 were checked with the polyclonal GFP antibody (Huaxin Bochuang, China). The PVDF membrane was stained with Ponceaux to visualize the large subunit of ribulose-1,5-bisphosphate as the loading control.

In human and *Arabidopsis*, SKP1/ASK1 interacts with CUL1 to assemble into SCF complexes through its N-terminal domain [37, 38]. We found that CLCuMuB βC1 interacts with N-terminal domain of NbSKP1.1 (Fig 2). This prompted us to investigate whether CLCuMuB βC1 interferes with the assembly of NbSKP1.1 into the SCF complex. To test this hypothesis, GFP competitive pull-down assay was performed. Because *E*. *coli-*expressed NbCUL1 was insoluble, GFP and GFP-tagged NbCUL1 (GFP-NbCUL1) were expressed in *N*. *benthamiana*, then precipitated by GFP-Trap beads. To eliminate the influence from endogenous NbSKP1s and NbSKP1L1, an excessive amount of *E*. *coli-*expressed His-HA-NbSKP1.1 was used to saturate the beads and endogenous NbSKP1s and NbSKP1L1 were crowded out from GFP-NbCUL1, then the supernatant was removed. After an increasing amount of *E*. *coli-*expressed His-tagged βC1 (His-βC1) was added, more and more His-HA-NbSKP1.1 was pulled off from GFP-NbCUL1. and levels of His-HA-NbSKP1.1 released into the supernatant were increased (Fig 3A).

Further, we confirmed CLCuMuB βC1 interfering with the interaction between NbSKP1.1 and NbCUL1 by BiFC assays. We generated nYFP-NbSKP1.1 and cYFP-NbCUL1 fusion constructs and co-expressed them with HA-tagged nLUC (HA-nLUC) or HA-tagged CLCuMuB βC1 (HA-βC1) in *N*. *benthamiana*. HA-βC1 is a functional protein (S3D–S3F Fig). Stronger signals were detected for the combination of nYFP-NbSKP1.1 and cYFP-NbCUL1 in the presence of HA-nLUC than in the presence of HA-βC1 (Fig 3B and 3C). Meanwhile, the protein level of nYFP-NbSKP1.1 and cYFP-NbCUL1 seem similar between the two groups (Fig 3D).

These data suggest that CLCuMuB βC1 interferes with the interaction between NbSKP1.1 and NbCUL1 via binding to NbSKP1.1.

### Silencing of *NbSKP1s* Enhances CLCuMuV Accumulation and Symptoms

βC1 but not βC1ΔC43 interacts with NbSKP1s and NbSKP1L1. Meanwhile βC1 but not βC1ΔC43 induces viral symptoms (S6 Fig). These results promote us to check whether silencing *NbSKP1s* can produce some viral symptoms. We constructed a deletion mutant betasatellite by replacing the entire *βC1* gene from CLCuMuB with sites of two restriction enzymes *Asc*I and *Xba*I to generate CLCuMuB (Δ*βC1*), hereafter called βM2 (S1 Fig). We guessed that our CLCuMuB-based vector βM2 may be used as a VIGS vector. To confirm this, we cloned a *N*. *benthamiana* phytoene desaturase (*NbPDS*) gene fragment into βM2 to generate βM2-*PDS*. Photo-bleach phenotype was observed around the leaf veins of *N*. *benthamiana* plants agroinoculated with βM2-*PDS* in the presence of helper virus CLCuMuV (S7 Fig). This result demonstrates that βM2 can be used as a CLCuMuB-based VIGS vector to effectively silence genes, and CLCuMuV may exhibit a phloem limitation.

**Fig 4:**
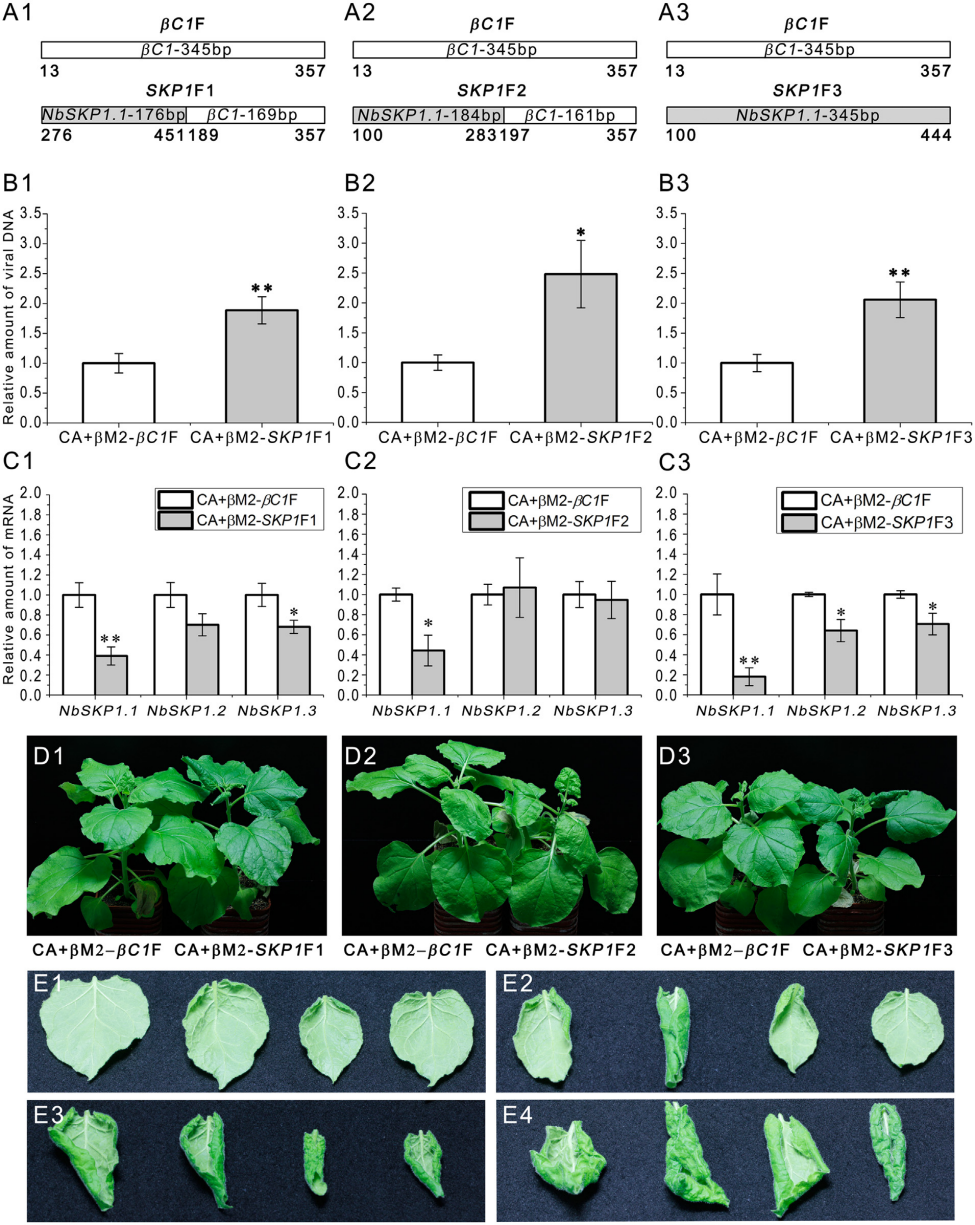
Silencing of *NbSKP1s* enhances CLCuMuV DNA accumulation and results in typical disease symptoms. (A1, A2 and A3) Six- to seven-week-old *N*. *benthamiana* plants were agroinoculated with CLCuMuV (CA) and βM2-*SKP1*F1 (A1), βM2-*SKP1*F2 (A2), βM2-*SKP1*F3 (A3) or βM2-*βC1*F (as the control). (B1, B2 and B3) Silencing of *NbSKP1s* enhanced CLCuMuV DNA accumulation. Each group contained 7 plants. At 14 dpi, total DNA was extracted from each plant respectively and subjected to quantitative real-time PCR (means±SEM, n = 7) to quantify viral DNA accumulation. The internal reference method was used to calculate the relative amount of viral DNA. (C1, C2 and C3) Real-time RT-PCR confirmed silencing of *NbSKP1s*. Total RNA was extracted from each plant respectively and subjected to quantitative RT-PCR (means±SEM, n = 4) to quantify *NbSKP1s* mRNA level. *Actin* was used as the internal reference. The raw data of (B1–B3) and (C1–C3) were analysed by two-sample *t*-test to show the significance level at 0.05 (*) and 0.01 (**). These experiments were repeated at least twice. (D1, D2 and D3) 50% plants infected with CA+βM2-*SKP1*F1 (D1), 50% plants infected with CA+βM2-*SKP1*F2 (D2) and 100% plants infected with CA+βM2-*SKP1*F3 (D3) show severe symptoms at 21 dpi. (E1, E2, E3 and E4) Apical leaves of plants infected with CA+βM2-*βC1*F (E1), CA+βM2-*SKP1*F1(E2), CA+βM2-*SKP1*F2 (E3) and CA+βM2-*SKP1*F3 (E4) at 21 dpi.

To investigate the role of *NbSKP1s* in CLCuMuV infection, we silenced *NbSKP1s* using our CLCuMuB-based VIGS vector, βM2. To exclude the effect from size, three cDNA fragments corresponding to the 176-bp, 184-bp and 345-bp *NbSKP1*.*1* sequences were fused with 169-bp, 161-bp and 0-bp *βC1* sequences respectively and then were cloned into βM2 to generate βM2-*SKP1*F1, βM2-*SKP1*F2 and βM2-*SKP1*F3 (Fig 4A1–4A3). A 345-bp fragment of *βC1* was inserted into βM2 to generate βM2-*βC1*F as the control. The position relationship among 176-bp, 184-bp and 345-bp *NbSKP1*.*1* fragments was shown in S8 Fig. *N*. *benthamiana* plants were agroinfiltrated with CLCuMuV (CA) and βM2-*βC1*F, βM2-*SKP1*F1, βM2-*SKP1*F2 or βM2-*SKP1*F3. Silencing of *NbSKP1s* resulted in an increasing accumulation of CLCuMuV DNA at 14 dpi (Fig 4B1–4B3). Since the mRNA level of *NbSKP1L1* was very low in normal plants (S9 Fig), and similar results can be found in the RNA-seq data of *N*. *benthamiana* in Sol Genomics Network (ftp://ftp.solgenomics.net/transcript_sequences/by_species/Nicotiana_benthamiana/), we gave up to check the mRNA level of *NbSKP1L1*. Silencing of *NbSKP1s* (*NbSKP1*.*1*, *NbSKP1*.*2* and *NbSKP1*.*3*) was triggered by all three constructs, and the levels of *NbSKP1s* mRNA were significantly reduced when compared to the βM2-*βC1*F control (Fig 4C1–4C3). βM2-*SKP1*F3 was more effective than βM2-*SKP1*F1 and βM2-*SKP1*F2 to cause silencing of *NbSKP1s* (Fig 4C1–4C3). At 21 dpi, 50% plants infected with CA+βM2-*SKP1*F1, 50% plants infected with CA+βM2-*SKP1*F2 and 100% plants infected with CA+βM2-*SKP1*F3 exhibited severe downward leaf curling and darkening as well as swollen veins, typical symptoms in plants infected by CA+β (Fig 4D1–4D3). If we continue to observe the symptom development, growth retardation will also be found (S10 Fig).

We also confirmed the effect of silencing *NbSKP1s* on CLCuMuV accumulation and symptoms using another control βM2-*GFP*F, which 345-bp *GFP* fragment was cloned into βM2. *N*. *benthamiana* plants were agroinfiltrated with CLCuMuV (CA) and βM2-*GFP*F or βM2-*SKP1*F3. We found again that silencing of *NbSKP1s* enhances CLCuMuV DNA accumulation and results in viral symptoms (S11 Fig).

TYLCCNB-based VIGS works mainly in vascular tissues [39], the tissues which CLCuMV tends to be limited to [40]. We further confirmed the effect of silencing *NbSKP1s* on CLCuMuV infection by TYLCCNB-based VIGS system [39]. We inserted the 345-bp *GFP* fragment and the 345-bp *SKP1*F3 fragment into pBinPLUS-2mβ of TYLCCNB-based VIGS system [39], then agroinoculated them respectively with TYLCCNV for silencing. Similarly, silencing of *NbSKP1s* enhanced CLCuMuV DNA accumulation and 100% *NbSKP1s* silenced plants exhibited viral symptoms (S12 Fig).

### Silencing of *NbCUL1* also Enhances CLCuMuV Accumulation and Symptoms

**Fig 5:**
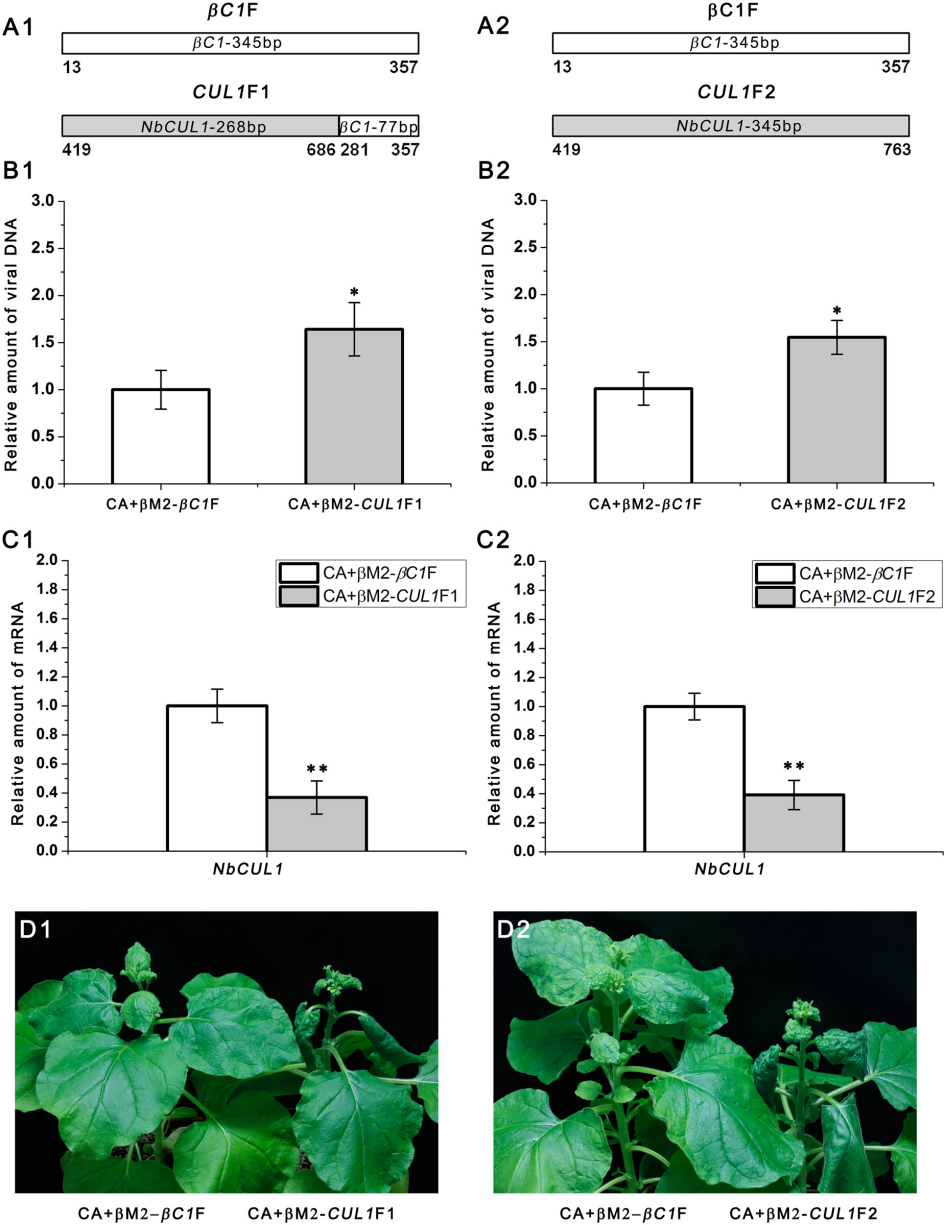
Silencing of *NbCUL1* enhances CLCuMuV DNA accumulation and results in typical disease symptoms. (A1 and A2) Six- to seven-week-old *N*. *benthamiana* plants were agroinoculated with CLCuMuV (CA) and βM2-*CUL1*F1 (A1), βM2-*CUL1*F2 (A2) or βM2-*βC1*F (as the control). (B1 and B2) Silencing of *NbCUL1* enhanced CLCuMuV DNA accumulation. Each group contained 7 plants. At 14 dpi, total DNA was extracted from each plant respectively and subjected to quantitative real-time PCR (means±SEM, n = 7) to quantify viral DNA accumulation. The internal reference method was used to calculate the relative amount of viral DNA. (C1 and C2) Real-time RT-PCR confirmed silencing of *NbCUL1*. Total RNA was extracted from each plant respectively and subjected to quantitative RT-PCR (means±SEM, n = 4) to quantify *NbCUL1* mRNA level. *Actin* was used as the internal reference. The raw data of (B1 and B2) and (C1 and C2) were analysed by two-sample *t*-test to show the significance level at 0.05 (*) and 0.01 (**). These experiments were repeated at least twice. (D1 and D2) 100% plants infected with CA+βM2-*CUL1*F1 (D1) or CA+βM2-*CUL1*F2 (D2) show severe symptoms at 21 dpi.

We have demonstrated that βC1 is able to interfere with the interaction between *NbSKP1s* and *NbCUL1* (Fig 3). Moreover, silencing of *NbSKP1s* has a dramatic influence on viral DNA accumulation and symptom development (Fig 4). We therefore investigated whether silencing of *NbCUL1* could also enhance CLCuMuV DNA accumulation and cause severe viral symptoms. Two cDNA fragments corresponding to the 268-bp and 345-bp sequences of *NbCUL1* were fused with 77-bp and 0-bp *βC1* sequences respectively and then were cloned into βM2 to generate βM2-*CUL1*F1 and βM2-*CUL1*F2 respectively (Fig 5A1 and 5A2). The position relationship among 268-bp, and 345-bp *NbCUL1* fragments were shown in S8 Fig. These two VIGS vectors along with CLCuMuV were then agroinfiltrated respectively into *N*. *benthamiana* plants. Silencing of *NbCUL1* by either CA+βM2-*CUL1*F1 or CA+βM2-*CUL1*F2 resulted in an higher accumulation of CLCuMuV DNA (Fig 5B1 and 5B2) and severer viral symptoms (Fig 5D1 and 5D2).

Taken together, these results suggest that βC1 may enhance its helper geminivirus’ accumulation and viral symptom induction by interfering with the interaction between SKP1 and CUL1 through its binding to SKP1.

### CLCuMuB βC1 Interferes with Hormone Signaling Pathways

**Fig 6:**
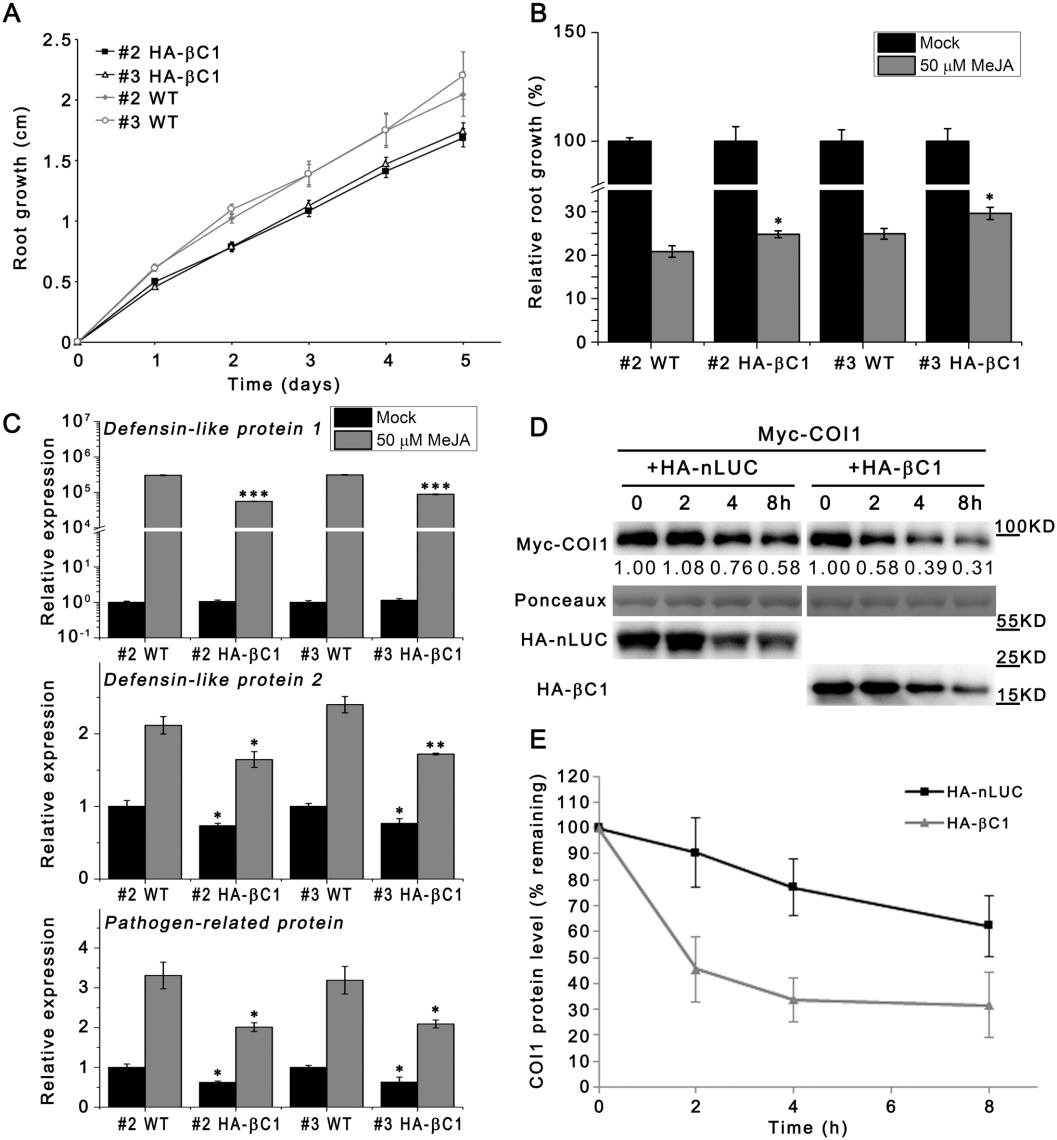
CLCuMuB βC1 represses JA responses though interfering with the integrity of SCF^COI1^. (A) Total root length of HA-βC1 transgenic (#2 HA-βC1 and #3 HA-βC1) and wild-type (#2 WT and #3 WT) *N*. *benthamiana* seedlings was measured every 24 h beginning at the 6th day after sowing (n ≥11). Bars represent SEM. #2 HA-βC1 and #2 WT were presented on same plates, while #3 HA-βC1 and #3 WT were presented on same plates. These experiments were repeated 3 times. (B) Jasmonate sensitivity was measured as root growth inhibition. Six-day-old seedlings (n ≥10) were grown on MS contained with 50 μM MeJA for additional 4 days. Bars represent SEM. The raw data were analysed by Mann-Whitney rank sum test to show the significance level at 0.05 (*). (C) Relative expression level of marker genes of jasmonate responses in mock- or MeJA-treated HA-βC1 transgenic and wild-type *N*. *benthamiana* seedlings determined by quantitative real-time PCR. #2 HA-βC1 and #2 WT were presented on same plates, while #3 HA-βC1 and #3 WT were presented on same plates. HA-βC1-expressing lines are compared with their corresponding control in each condition. *Actin* was used as the internal control. Bars represent SEM. The raw data were analysed by two-sample *t*-test to show the significance level at 0.05 (*), 0.01 (**) and 0.001 (***). These experiments were repeated at least twice. (D) CLCuMB βC1 enhanced degradation of COI1 *in vitro*. The purified Myc-COI1 protein was added to total protein extracts from *N*.*benthamiana* which expressed HA-nLUC or HA-βC1, incubated at 25°C for the indicated time periods, and subjected to immunoblot analysis with the anti-Myc antibody. Intensity was detected through Total Lab TL120. The PVDF membrane was stained with Ponceaux to visualize the large subunit of ribulose-1,5-bisphosphate as the loading control. (E) Quantitative analysis of the relative abundance of COI1 in the presence of HA-nLUC or HA-βC1 for the time periods indicated. The abundance of COI1 at the start point (0-h) was set to 100% as a reference for calculating its relative abundance after different incubation periods. Error bars represent SD. The experiment was repeated three times.

Because βC1 interferes with the interaction between SKP1 and CUL1, and *cul1* mutants are altered in JA responses [41, 42], we tested whether βC1 can interfere with JA pathways. First, we evaluated root growth rate in HA-βC1 transgenic plants, the root length of 6-day-old seedlings was measured every 24 h for 5 days. Data showed that HA-βC1 transgenic roots grow more slowly than wild-type roots (Fig 6A). Meanwhile, we measured inhibition of primary root elongation caused by treatment with methyl-jasmonate (MeJA), and HA-βC1 transgenic plants showed less sensitivity than wild-type plants to 50 μM MeJA (Fig 6B). Further, quantitative real-time PCR was used to quantify the mRNA level of marker genes for JA responses. Three genes: *Defensin-like protein 1*, *Defensin-like protein 2* and *Pathogen like protein* were chosen for JA responses. Compared to wild-type plants, all three markers genes showed lower mRNA expression level in two independent HA-βC1 transgenic lines (#2 HA-βC1 and #3 HA-βC1) (Fig 6C). Auxin and gibberellins signalings are also regulated by CUL1-based SCF ubiquitin E3 ligases [27, 29]. Real-time PCR assays showed lower mRNA expression level of their marker genes (*Gibberellin-regulated protein 14* and *Gibberellin-regulated protein 6* for gibberellins, *SAUR14* and *PID* for auxin) in HA-βC1 transgenic lines than in wild-type controls (S13A and S13B Fig).

Taken together, CLCuMuB βC1 can really cause deficient function in SCF complexes and interfere with hormone signaling pathways.

### CLCuMuB βC1 Does Not Hinder JA Biosynthesis but Interferes with the SCF^COI1^ Function

SCF^COI1^ is the receptor for JA, and some geminiviruses interfere with JA pathway [20, 21, 33, 43, 44]. Meanwhile CLCuMuB βC1 seems to have no inhibition on jasmonates biosynthesis according to JA level data measured by mass spectrum and HPLC. Regardless of being wounded or not, plants infected with CA+β showed higher JA level compared to plants infected with CA+βM1 or healthy plants (S14 Fig). These results imply that CLCuMuB βC1 doesn’t impair JA biosynthesis. Higher JA level in plants infected with CA+β may be derived from the feedback due to the impaired JA signaling.

The stability of JA receptor COI1, a F-box protein, is dependent on an intact SCF^COI1^ complex [45]. Because βC1 can interfere with the interaction between SKP1 and CUL1, we assumed that it may reduce the stability of COI1 *in vitro*. Co-IP analysis indicated that GFP-CUL1 associated with both Myc-COI1 and HA-NbSKP1.1 (S15 Fig), suggesting that Myc-COI1 can be integrated within SCF complexes. After Myc-COI1 was transiently expressed in *N*.*benthamiana* and purified with anti-Myc affinity beads. Myc-COI1 protein was then mixed with total protein extracts prepared from *N*.*benthamiana* which was transiently expressed HA-βC1 or HA-nLUC. The stability of Myc-COI1 was assessed by western blot assays after the treatment at 25°C for various periods of time up to 8 h. The Myc-COI1 protein degraded more rapidly in HA-βC1 extracts compared to in HA-nLUC extracts (Fig 6D and 6E). Moreover, the accumulation of Myc-COI1 in HA-βC1 transgenic lines was reduced 84–92% compared to that in wild-type plants (WT) (S16 Fig), whilst the accumulation of GFP (as an expression control) in HA-βC1 transgenic lines was reduced by 26–41% in WT plant (S16 Fig).

Taken together, these data implied that CLCuMuB βC1 damages the integrity of SCF^COI1^ complex to hinder JA responses.

### CLCuMuB βC1 also Hinders the Degradation of GAI, Target of the SCF^SLY1^ *In Vivo*

**Fig 7:**
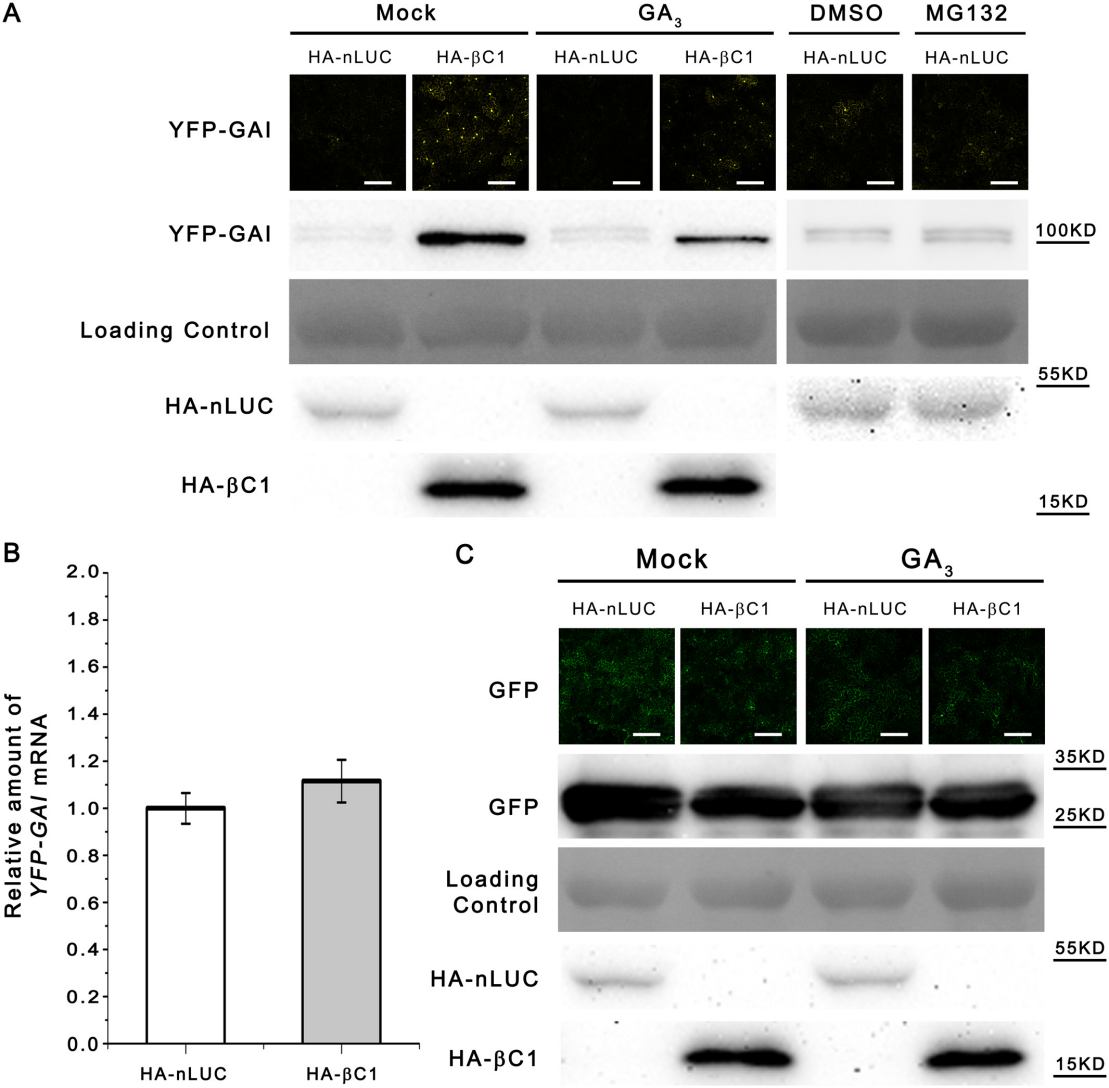
CLCuMuB βC1 hinders the degradation of YFP-GAI *in vivo*. (A) CLCuMuB βC1 attenued degradation of YFP-GAI *in vivo*. YFP-GAI expression construct was coinfiltrated with constructs expressing HA-nLUC or HA-βC1 into seven to eight-week-old *N*. *benthamiana* plant leaves. Around 48 hpi, agroinfiltrated leaves were sprayed with 100 μM GA_3_ or mock solution (ethonal) and visualized via a Zeiss LSM 710 laser scanning microscope. Bar scales represents 200 μm. DMSO and MG132 (50 μM) were applied into plant leaves 12 h before observation. Protein level was analyzed via SDS-PAGE and western blot analysis with the anti-GFP antibody, which also recognizes YFP. The PVDF membrane was stained with Ponceaux to visualize the large subunit of ribulose-1,5-bisphosphate as a loading control. (B) Real-time RT-PCR detected the mRNA level of YFP-GAI. Total RNA was extracted from each *N*. *benthamiana* leaves and then subjected to quantitative RT-PCR (means±SEM, n = 3) to quantify YFP-GAI mRNA level. *eIF4a* was used as the internal reference. (C) CLCuMuB βC1 didn’t affect stability of GFP *in vivo*. Detection of GFP (as an internal control) in *N*. *benthamiana* leaves coinfiltrated with the construct expressing GFP together with constructs expressing HA-nLUC or HA-βC1 and treated with 100 μM GA_3_ or mock (ethanol) solution and visualized via a Zeiss LSM 710 laser scanning microscope. Bar scale represents 200 μm. Protein level was analyzed via SDS-PAGE and immunoblot analysis with anti-GFP. The PVDF membrane was stained with Ponceaux to visualize the large subunit of ribulose-1,5-bisphosphate as a loading control.

GA releases the brakes of plant growth. During this process, DELLA protein GAI is ubiquitinated by the SCF^SLY1^ and eventually degradated by the 26S proteasome [46]. Mutant plants that are deficient in GA pathways exhibit a dwarf phenotype [46]. Further, plants infected with CA+β is dwarf compared to plants infected with CA+βM1 (S2 Fig). To check whether the function of SCF^SLY1^ is hindered by CLCuMuB βC1, we co-expressed YFP-GAI with either HA-βC1 or HA-nLUC to investigate its degradation as described [33]. At 48 hpi, YFP-GAI fluorescence was observed in the nuclei 48 hpi (Fig 7A), indicating YFP-GAI can be co-expressed with HA-βC1 or HA-nLUC normally in *N*. *benthamiana* leaves. However, whether plants were treated with 100 μM GA_3_ or not, YFP-GAI fluorescence was enhanced when co-expressed with HA-βC1 (Fig 7A). Western blot assays using an anti-GFP antibody indicated that YFP-GAI accumulation was less in plants co-expressed with HA-nLUC than those co-expressed with HA-βC1 (Fig 7A). Meanwhile, co-expression with HA-βC1 or HA-nLUC did not significantly affect mRNA level of YFP-GAI at this time point (Fig 7B). Moreover, co-expression of HA-βC1ΔC43 did not enhance YFP-GAI accumulation (S17 Fig). As an internal control, a GFP expression construct was coinfiltrated with HA-βC1 or HA-nLUC expression construct. No significant differences in GFP fluorescence or GFP protein accumulation were detected between them (Fig 7C).

Taken together, these results indicate that CLCuMuB βC1 can increase the accumulation of GAI by hindering its degradation to hinder GA responses.

### Exogenous MeJA Treatment Reduces Plant Susceptibility to CLCuMuV

**Fig 8:**
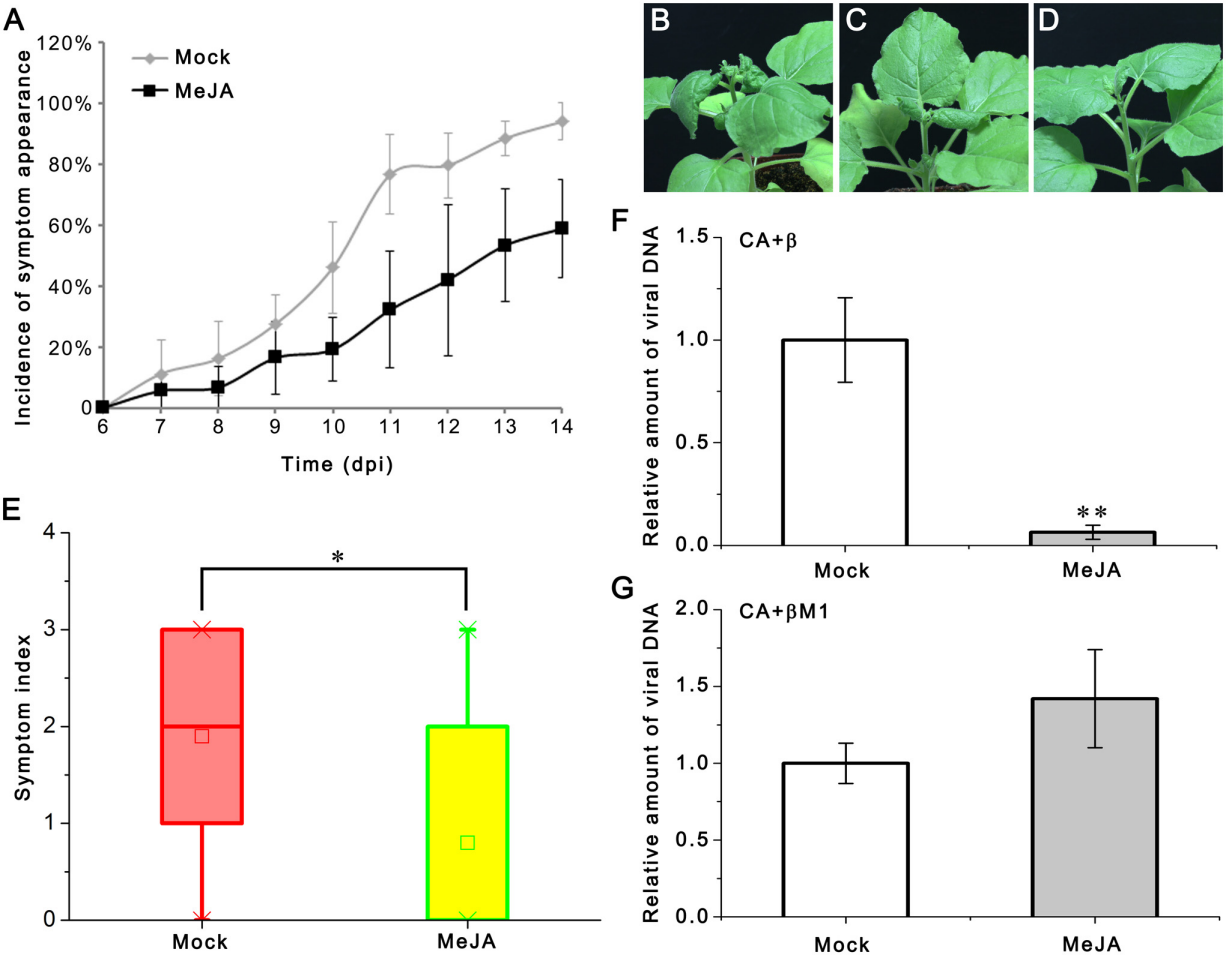
JA treatment enhances plant defense against CLCuMuV. (A) Exogenous MeJA treatment delayed the incidence of symptom appearance. Six- to seven-week-old *N*. *benthamiana* (10–12 plants per treatment) were agroinoculated with CA+β, treated every other day with 50 μM MeJA or mock solution (ethonal), and recorded for the symptom appearance until 14 dpi. Means came from three independent experiments, Error bars represent SEM. (B-D) Different levels of symptoms (B: the 4^th^ level, C: the 2^nd^ level, and D: level 0 showing no symptom). (E) Exogenous MeJA treatment attenued disease symptoms level. Plants were scored for the appearance of symptoms at 14 dpi. (F) Total DNA was extracted from each plant respectively and subjected to real-time PCR (means±SEM, n = 7) to quantify viral DNA accumulation at 14 dpi. (G) Six- to seven-week-old *N*. *benthamiana* (10 plants per treatment) were agroinoculated with CA+βM1, treated every other day with 50 μM MeJA or mock solution (ethonal), Total DNA was extracted from each plant respectively and subjected to real-time PCR (means±SEM, n = 7) to quantify viral DNA accumulation at 14 dpi. The internal reference method was used to calculate the relative amount of viral DNA. The raw data of (E, F and G) were analysed by two-sample *t*-test to show significance level at 0.05 (*) and 0.01 (**). These experiments were repeated at least twice.

βC1 interferes with SCF function to enhance geminivirus DNA accumulation and damages the integrity of SCF^COI1^ complex to hinder JA responses. This would suggest that JA is likely to be involved in plant defense against CLCuMuV. To test this hypothesis, we inoculated CLCuMuV along with CLCuMuB into MeJA or mock-treated *N*. *benthamiana* plants. Symptoms were daily monitored from 9 to 14 dpi. We found that application of exogenous MeJA resulted in milder symptoms (Fig 8A–8E) and lower viral DNA accumulation (Fig 8F). These results demonstrate that MeJA could compromise viral pathogenicity. We also inoculated CLCuMuV along with βM1 into MeJA or mock-treated *N*. *benthamiana* plants. Real-time results show no difference on viral DNA accumulation between the two kinds of treatment (Fig 8G). Thus, βC1 may enhance geminivirus infection, at least partially by inhibiting JA pathway through interfering with the function of SCF^COI1^.

## Discussion

In this study, we found that CLCuMuB βC1 inhibits the function of SCF ligase to enhance geminivirus DNA accumulation and symptom development by disrupting SKP-CUL1 interaction through its binding to SKP1. In addition, we found that JA treatment improves plant defense against geminivirus infection.

### Molecular Basis of Virus Symptoms Elicited by Geminivirus βC1

Betasatellites are indispensable for some monopartite geminiviruses to induce viral symptoms in host plants. The sole protein βC1 encoded by several betasatellites, has been reported to be responsible for this phenomenon [1]. However, how βC1 induces viral symptoms remain obscure. CLCuMuB βC1 was previously reported to interact with a tomato ubiquitin conjugating enzyme (UBC), SlUBC3, by its C-terminal myristoylation-like motif [22]. The myristoylation-like motif only exists in CLCuMuB βC1 and its close relative *okra leaf curl betasatellite* (OLCB βC1). However, OLCB βC1 does not interact with SlUBC3 [22]. Further, silencing of *UBC3* in *N*. *benthamiana* did not cause any obvious phenotype and enhanced viral DNA accumulation in this study (S18 Fig). Thus, it is possible that symptoms induced by CLCuMuB might not be mediated by interaction between βC1 proteins and host UBC3 enzyme. Here, we demonstrate that CLCuMuB βC1 is also indispensable for symptom production (S2 Fig). Through a series of interaction assays, we found that CLCuMuB βC1 interacts with NbSKP1s, important components of SCF complexes (Fig 1). Further, CLCuMuB βC1 interferes with the interaction between SKP1 and CUL1 (Fig 3) to impair the function of SCF complexes, such as SCF^COI1^ and SCF^SYL1^ (Figs 6 and 7), which is consistent with the previous observation that overexpression of CLCuMuB βC1 in tobacco causes a global reduction of polyubiquitinated proteins [22]. We found that disrupting the function of SCF complexes by silencing of either *SKP1* or *CUL1* leads to some typical virus symptoms, such as severe leaf curling, crimping, leaf darkening and growth retardation (Figs 4 and 5). Indeed, perturbation of the ubiquitin system can cause leaf curling and vascular tissue abnormalities [47]. Further, overexpression of CLCuMuB βC1 blocked the degradation of GAI (Fig 8), the target of the SCF^SLY1^, repressed plant responses to GA, which may explained why the presence of CLCuMuB make plant dwarf phenotype. These results suggest that some geminiviral βC1 proteins can elicit viral symptoms by disrupting the plant ubiquitination pathway by interfering with SKP1-CUL1 interaction through its interaction with SKP1.

Although *NbSKP1s* silencing is in fact causing higher accumulation of viral DNA (Fig 4B1–4B3), the symptoms seem simply due to *NbSKP1s* silencing but not higher accumulation of virus, because we found higher accumulation of CLCuMuV DNA, but no symptom in plants infected with CLCuMuV and βM2-*SKP1*-176 which is generated though inserting the 176-bp *NbSKP1*.*1* fragment directly into βM2, without fused with the 169-bp *βC1* fragment (S19 Fig).

We noticed that silencing of either *SKP1* or *CUL1* did not produce all symptoms caused by CLCuMuB βC1. Besides leaf curling, crimping, darkening and growth retardation caused by silencing of either *SKP1* or *CUL1*, the viral symptoms elicited by CLCuMuB βC1 also include bending shoot and enations from abaxial side of leaves. *Tomato yellow leaf curl China virus* (TYLCCNV) βC1 was reported previously to elicit leaf morphological changes in *Arabidopsis* by mimicking the functions of ASYMMETRIC LEAVES 2 through its interaction with ASYMMETRIC LEAVES 1 and by repressing the accumulation of miR165/166 to subvert leaf polarity [20]. Meanwhile, suppression of miR165/166 can cause enations from abaxial side of leaves [48]. It is possible that CLCuMuB βC1 induces enations by suppression of miR165/166. Further, TYLCCNV βC1 may also induce viral symptoms by up-regulating the expression of a calmodulin-like protein (rgsCaM) [16]. Considering that geminivirus βC1 is a multiple functional protein, CLCuMuB βC1 may contribute to the viral symptoms by multiple mechanisms including disrupting the plant ubiquitination pathway.

### Molecular Basis of Geminivirus βC1 Enhancing Virus Accumulation

In this study, we demonstrate that CLCuMuB βC1 impairs the interaction between NbSKP1s and NbCUL1 by interacting with NbSKP1s and silencing of either *NbSKP1s* or *NbCUL1* enhances CLCuMuV DNA accumulation. Deletion of CLCuMuB βC1 reduced CLCuMuV titer (S2 Fig). Silencing of either *NbSKP1s* or *NbCUL1* caused enhanced virus accumulation (Figs 4 and 5). Geminiviruses may interfere with plant ubiquitination to suppress plant defense against geminivirus infection [49]. It has been reported that V2 protein of *Tomato yellow leaf curl Sardinia virus* (TYLCSV) interacts with UBA1, a ubiquitin-activating enzyme, which is a positive regulator of plant defense [50, 51], and silencing of either *UBA1* or *RHF2a* (RING-type E3 ubiquitin ligase) in *N*. *benthamiana* enhances TYLCSV infection [50, 52]. Geminiviral C4 activates expression of host RING E3 ligase RKP to ubiquitinate cell cycle inhibitors ICK/KRPs to help the replication of *Beet severe curly top virus* (BSCTV) via promoting cell division [53, 54]. However, how geminivirus βC1 proteins interfere with plant ubiquitination pathway to enhance viral accumulation is still obscure.

In this study, we found that CLCuMuB βC1 disrupted the integrity of SCF^COI1^ (Fig 6D and 6E). Meanwhile CLCuMuB βC1 does not inhibit JA biosynthesis (S14 Fig). More importantly, JA treatment reduces the plant susceptibility to CLCuMuV (Fig 8), which is consistent with the previous observation that JA treatment attenuates the infection of plant with *Beet curly top virus* (BCTV) [33]. TYLCCNB βC1 was reported to suppress JA-related host defenses for increasing population densities of their whitefly vectors [19, 21]. Further, *Cabbage leaf curl virus* (CaLCuV) infection can also repress JA response [21, 44]. The C2 proteins of TYLCSV, *Tomato yellow leaf curl virus* (TYLCV) and BCTV were reported to impair derubylation of SCF E3 ligase complexes and inhibit jasmonate signaling by interacting with CSN5 [20, 33]. Thus, CLCuMuB βC1 could enhance CLCuMuV accumulation, at least partially by repressing JA responses through interfering with plant ubiquitination.

We observed that the levels of CLCuMuV DNA in *SKP1-* or *CUL1-*silenced plants were lower than that in the presence of CLCuMuB with functional βC1 although silencing of either *SKP1* or *CUL1* resulted in a higher accumulation of CLCuMuV DNA (Figs 4 and 5 and S2). It has been reported that knock-down of either CSN5A or CSN3, two components of protein degradation-related CSN complexes, hinders BCTV infection although knockout of *Arabidopsis csn5a* mutant can partially complement BCTV C2 mutant [50, 52, 55]. Further, overexpression of a given F-box protein can circumvent the general SCF malfunction [56, 57]. These observations suggest that begomoviruses might not only hamper, but also redirect the activity of SCF complexes for begomoviruses propagation [33]. Very recently, ubiquitination is reported to regulate the stability of TYLCCNV βC1 [58]. Thus, host plants, geminiviruses and their satellites may have evolved to exploit the dual roles of the ubiquitination pathway in plant defense and viral pathogenesis to co-survive in their long-term arm races.

## Methods

### Plasmid Construction

The full-length infectious CLCuMuV clone contains 1.7-mer CLCuMuV DNA genome. Two separate DNA fragments were PCR amplified using primer pairs *Hind*III-A-F/*Xba*I-A-R, or *Xba*I-A-F/*Kpn*I-A-R respectively and total DNA extracted from cotton leaf tissues with CLCuD [34] as the template, double-digested with *Hind*III and *Xba*I or *Xba*I and *Kpn*I, and then inserted into pBinplus ARS digested with *Hind*III and *Kpn*I.

The βDNA infectious clone contains 2-mer CLCuMuB genomes. Two DNA fragments were PCR amplified using primer pairs *Kpn*I-β-F/*Hind*III-β-R or *Hind*III-β-F/*Sac*I-β-R respectively and total DNA from cotton samples with CLCuD [34] as the template, digested with *Kpn*I and *Hind*III or *Hind*III and *Sac*I, and then inserted into pCAMBIA-2300 digested with *Kpn*I and *Sac*I to generate βDNA.

The null mutant betasatellite vector βM1 was constructed by introducing a ATG-TGA transition in the start codon. βDNA was used as the template. Two DNA fragments were PCR amplified using primer pairs βM1-R/*Sac*I-β-R or *Hind*III-β-F/βM1-F respectively, then were fused to obtain *Sac*I-βM1-*Hind*III with ATG-TGA mutation. the other two DNA fragments were PCR amplified using primer pairs *Hind*III-β-F/βM1-R and βM1-F/*Kpn*I-β-F, then were fused to obtain *Hind*III-βM1-*Kpn*I with ATG-TGA mutation. digested with *Sac*I and *Hind*III or *Hind*III and *Kpn*I, *Sac*I-βM1-*Hind*III and *Hind*III-βM1-*Kpn*I were inserted into pCAMBIA-2300 digested with *Kpn*I and *Sac*I to generate βM1.

The T-DNA silencing vector βM2 was constructed by introducing a multiple cloning site to replace the *βC1* ORF in CLCuMuB. Two DNA fragments were PCR amplified using primer pairs *Kpn*I-βMF/*Xba*I-βM2-R or *Xba*I-βM2-F/*Sac*I-βM2-R respectively using βDNA as the template, digested by *Kpn*I and *Xba*I or *Xba*I and *Sac*I, and then inserted into pCAMBIA-2300 digested by *Kpn*I and *Sac*I to generate vector βM2.

DNA fragments of HA-βC1-nYFP, HA-βC1ΔC43-nYFP, HA-cYFP-NbSKP1.1, HA-cYFP-NbSKP1L1, HA-cYFP-nLUC, GFP-βC1, HA-βC1, HA-βC1ΔC43, HA-NbSKP1.1, GFP-NbCUL1, nYFP-SKP1, cYFP-NbCUL1, Myc-COI1 and YFP-GAI were obtained by overlapping PCR. The resulting PCR products were cloned between the duplicated *Cauliflower mosaic virus* 35S promoter and Nos terminator of pJG045, a pCAMBIA1300-based T-DNA vector [59]. βC1pro:βC1, a βC1expression vector with its native promoter, was generated by inserting 1–1346 nt of CLCuMuB genome (GQ906588) into pCAMBIA-2300. Among these vectors, βC1pro:βC1, *35Spro*:*GFP-βC1* and *35Spro*:*HA-βC1* were used to generate transgenic plants respectively. PVX-cLUC, PVX-βC1 and PVX-βC1ΔC43 were constructed by introducing DNA fragments of cLUC, βC1 and βC1ΔC43 into a PVX vector [60]. pBinPLUS-TA and pBinPLUS-2mβ were kindly provided by Professor Xueping Zhou [61]. All constructs were confirmed by DNA sequencing. Primers used in this study were listed in S1 Table.

### Quantification of Viral DNA

Total DNA was extracted from apical developing leaves using the DNAsecure Plant Kit (TIANGEN, China). DNA concentration of each sample was calculated through OD_260_ via Epoch Multi-Volume Spectrophotometer System (Bio-Tek, USA) and then diluted to around 60ng/ul for PCR amplication. A single copy of CLCuMuV genome was amplified by PCR and then was ligased into pMD19-T (TaKaRa, Japan) to generate a CLCuMuV-positive plasmid. A 10-fold serial dilution of the plasmid DNA from 2×10^8^ to 200 copy was prepared and used as the standard. A CLCuMuV-specific primer set (qCLCuMuV V1-F and qCLCuMuV V1-R) was used to amplify a 198-bp amplicon. For SYBR Green-based real-time PCR performed in a 10 μL reaction mixture containing 5 μl Power SYBR Green PCR Master Mix (2×) (Life, USA), primer concentration was optimized by running the assay using the plasmid DNA dilution series with two different primer concentration (10 and 20 μM). 0.1 μL of each 20 μM primer and 0.3 μL 60 ng/μL templet were finally chosen to amplify viral DNA in samples for following assays. Because the standard curves generated were linear in the whole range tested with a coefficient of regression R^2^:0.99 and calculated slope around -3.5 for SYBR Green assay. The copy number of viral DNA can be calculated via Ct value of each sample and the standard curve.

To obtain the ratio of viral DNA: plant genome DNA, Plant genome DNA can also be calculated via internal reference method. The genome DNA of healthy *N*.*benthamiana* was extracted and a 2-fold serial dilution of the genome DNA from 94.5ng to 1.48ng was prepared and used as the standard. An *eIF4a*-specific primer set (q*eIF4a*-F and q*eIF4a*-R) was used to amplify a 60-bp amplicon. Primer concentration was optimized by using the plant genome DNA dilution series with three different primer concentrations (10, 15 and 20 μM). 0.1 μL of each 15 μM primer was finally chosen because the standard curves generated were linear in the whole range tested with a coefficient of regression R^2^:0.99 and calculated slope around -3.3 for SYBR Green assay. The plant genome DNA can be calculated via Ct value of each sample and the standard curve.

### Yeast Two-Hybrid Screen and Interaction Assays

The full-length CLCuMuB *βC1* was PCR amplified and cloned into yeast vector pYL302 to generate the LexA DNA binding domain (BD) containing bait vectors BD-CLCuMuB *βC1*. The full-length *NbSKP1*.*1*, *NbSKP1*.*2*, *NbSKP1*.*3*, *NbSKP1L1* and *NbSKP1*.*1* deletion derivatives were PCR amplified and cloned into the B42 activation domain (AD)-containing vector pJG4-5. The yeast two-hybrid prey library containing tomato cDNAs was used to screen CLCuMuB βC1-binding proteins. The yeast two-hybrid screen and interaction assays were performed as described [35].

### Plant Growth and Agroinfiltration

*N*. *benthamiana* plants were grown in pots at 25°C in growth rooms under 16 h light/8 h dark cycle with 60% humidity. Light intensity is 4000 lx. Solt mixed with vermiculite at a 1:1 ratio was used as the substrate for plants to grow. the plants were watered with a nutrient solution.

For CLCuMuB-based VIGS assays, CLCuMuV or βM2 and its derivatives were introduced into *Agrobacterium* strain GV2260. *Agrobacterium* cultures containing CLCuMuV or βM2 derivative plasmids were grown overnight at 28°C until OD_600_ = 2.0, then CLCuMuV with corresponding βM2 derivative vector were mixed at 1: 1 ratio, pelleted, resuspended in infiltration buffer (10 mM MgCl_2_, 10 mM MES, and 200 μM acetosyringone, pH 5.6) to OD_600_ = 1.0, kept at room temperature for 4 h and infiltrated into the lower leaf of 6-leaf stage plants using a 1-ml needleless syringe.

For *Agrobacterium tumefaciens*-mediated transient expression studies, GV2260 strains containing the relevant expression vectors were cultured and prepared as described above, then were infiltrated into *N*. *benthamiana* leaves. The infiltrated leaves were detached at 48 to 60 hpi for the corresponding assays. For coexpression, equal amounts of *A*. *tumefaciens* cultures were mixed and used for infiltration.

MeJA treatments: a 50 μM MeJA solution or mock solution (ethanol) were applied to 6-week-old *N*. *benthamiana* plants by spray every other day from 1 day before the inoculation to 14 dpi.

### BiFC and Fluorescence Microscopy

Citrine YFP-based BiFC was performed as described [36]. The experimental group and corresponding control group should be inoculated in a same leaf to reduce the difference of expression condition. Live plant imaging was performed on a Zeiss LSM710 confocal microscope. Enhanced citrine YFP-derived fluorescence was acquired using 514-nm laser and emission 519- to 587-nm filters. 8-bit confocal images were acquired with an EC Plan-Neofluar 103/0.30 M27 objective for 103 magnification and a Plan-Apochromat 403/0.95 Korr M27 objective for 403 magnification. Images were analyzed with ZEN 2012 Light Edition.

### Quantification of YFP Fluorescence Intensity

The experimental group and corresponding control group were inoculated in a same leaf. At 48 dpi, images of live plant samples from experimental and corresponding control groups were taken under the same parameters via a Zeiss LSM710 confocal microscope. Software ZEN 2012 was used to measure the fluorescence intensity mean value of an image. 4 independent images for each group were measured and values were analyzed via *t*-test. Three biological repeats were needed.

### Co-immunoprecipitation (Co-IP)

Because βC1 protein was reported not stable *in vivo* and may be degraded through ubiquitin 26S proteasome system (UPS) [20], so in this assay we added MG132, an inhibitor against the 26S proteasome, to improve the accumulation of GFP-βC1. For Co-IP assays, 50 μM MG132 (Sigma, USA) was inoculated into *N*. *benthamiana* leaves 12 h before being detached. total proteins from leaves were extracted with a ratio of 1:2 of native extraction buffer 1 [NB1; 50 mM TRIS-MES pH 8.0, 0.5 M sucrose, 1 mM MgCl_2_, 10 mM EDTA, 5 mM DTT, 50 μM MG132, protease inhibitor cocktail CompleteMini tablets (Roche, http://www.roche.com/)] [62]. Protein extracts were incubated with the GFP-Trap beads (ChromoTek, German) for 2 hours at 4°C, The beads were washed three times with ice-cold NB1 at 4°C. IP samples were analyzed by SDS-PAGE, immunoblotted using anti-HA (CST, USA) and anti-GFP antibodies (Abmart, China) and detected using Pierce ECL western blotting substrate (Thermo, USA).

### GST Pull-Down Assay

GST-CLCuMuB βC1 and HA-His-NbSKP1.1 fusion proteins were produced in BL21(DE3) codon plus RIL cells. HA-His-NbSKP1.1 was purified using Ni-NTA Agarose (Qiagen, Netherlands) column. GST-CLCuMuB βC1 was purified using Glutathione Sepharose 4B (GE, USA) and then used to pull down HA-His-NbSKP1.1 *in vitro* for 2 hours at 4°C. The beads were washed three times with ice-cold elution buffer (300 mM NaCl, 50 mM Tric-HCl, pH 8.0, 0.1% Triton-X 100) at 4°C. The washed beads were boiled in SDS sample buffer, and proteins were separated by SDS-PAGE and detected by western blot using an anti-HA antibody(CST, USA).

### GFP Competitive Pull-Down Assay

His-CLCuMuB βC1 and HA-His-NbSKP1.1 fusion proteins were produced in BL21(DE3) codon plus RIL cells. *E*. *coli* cells harboring the corresponding clones were cultured in LB medium (5 mL) containing kanamycin (50 μg/mL) at 37°C, till the O.D. at 600 nm reached 0.6. Then the cells were inoculated for large scale expression. The expression of corresponding genes were induced by the addition of isopropyl-β-D-thiogalactopyranoside (IPTG, Sigma) to the final concentration of 0.2 mM and cells were further allowed to grow for 20 hours at 16°C. The cells were spun down at 4,000 rpm, resuspended in the ice-cold lysis buffer (50 mM Tris-HCl, 300 mM NaCl, 1 mM PMSF, 50 mM DTT, pH 8.5). Resuspended cells were sonicated till suspension became optically clear. HA-His-NbSKP1.1 was soluble and purified using Ni-NTA Agarose (Qiagen, Netherlands) column. His-CLCuMuB βC1 was in inclusion bodies and was dissolved by 8 M Urea (50 mM DTT, 8 M Urea) with a ratio of 0.1g: 1ml. Insoluble substance were removed by centrifugation at 14,000 rpm, 30 min, 4°C. Supernatant was dripped slowly using a 1-ml syringe with needle into 200 mL ice-cold refolding buffer (50 mM Tris-HCl, 300 mM NaCl, 500 mM Arginine, 2 M Urea, 1 mM PMSF, pH 8.5) agitated by a magnetic stirring apparatus. Then this His-CLCuMuB βC1 solution was dialyzed against the dialysis buffer (50 mM Tris-HCl, 300 mM NaCl, pH 8.5). The protein obtained by this method was enriched by Ni-NTA Agarose (Qiagen, Netherlands) column and eluted for further experiments.

1 mL GFP-CUL1 or GFP extracts were prepared and immunoprecipitated by 20 μL GFP-Trap beads (ChromoTek, German) for each sample as described in the Co-Immunoprecipitation (Co-IP) part. After two washes with wash buffer (50 mM Tris-HCl, 300 mM NaCl, 1 mM PMSF, 50 mM DTT, pH 8.5), 1 mL 100 μg/mL *E*. *coli-*expressed His-HA-NbSKP1.1 was added and incubated at 4°C for 1 hour. After two washes with wash buffer, 80 μg, 40 μg, 20 μg His-βC1 or 80 μg BSA was added in 1 mL corresponding samples and incubated at 4°C for 1 hour. After three washes with wash buffer, samples were separated by SDS-PAGE, transferred to PVDF membrane, and detected with corresponding antibodies.

### Root Growth and Jasmonate Inhibition Assays

The experiments were performed as described by Lozano-Duran [33]. Seeds of wild-type or HA-βC1 transgenic *N*. *benthamiana* used in this study were surface sterilized and sown on Murashige and Skoog (MS) agar plates with 30 g/L sucrose and 0.6% Agar. Seedlings were grown at 25°C under 4000 lx white light with a 16-h-light/8-h-dark photoperiod. MS plates were placed in a vertical orientation for 6 d, and seedlings were then transferred to MS plates containing no or 50 μM MeJA (Sigma, USA). Root length was scanned every day until 5 days later.

### JA Level Analysis

14–15 days *Nicotiana benthamiana* plants were inoculated with CA+β or CA+βM1. Leaves in three replicate plants for each treatment were sampled. The leaf materials from each plant were flash-frozen in liquid nitrogen, weighed and stored at -80°C until JA analysis. Sample preparation was performed as described by Glauser and Wolfender, [63]. Except methanol–water, 40:60 (v/v) was used to resolubilize the final residue and do subsequent UHPLC-Q-TOFMS Analysis. Drug Discovery Facility, Center of Biomedical Analysis, Tsinghua University provided the service for sample determination.

### *In Vivo* GAI Degradation Assay Analysis

GAI was cloned from cDNA of *N*. *benthamiana* and the experiments were performed as described by Lozano-Duran [33]. At 48 h past inoculation, the agroinfiltrated leaves were sprayed with a 100 μM GA_3_ solution or with mock solution (ethanol). Fluorescence was visualized 1 to 2 hours later using a Zeiss LSM710 confocal microscope. Leaf samples were grind by liquid nitrogen, Then total proteins were extracted with a ratio of 1:4 of extraction buffer (50 mM Tris-HCl, 100 mM NaCl, 25 mM imidazole, 10% glycerol, 0.1% Tween-20, 20 mM β-mercaptoethanol) [45]. Samples were separated by SDS-PAGE, transferred to PVDF membrane, and detected with the anti-GFP (ChromoTek, German).

### *In Vitro* COI1 Degradation Assay Analysis

Myc-COI1 was expressed in *N*. *benthamiana* and purified as described [45]. 60 μL of purified protein was added to 540 μL of total crude protein extracts (1 mg/mL) from *N*.*benthamiana* which was transiently expressed HA-βC1 or HA-nLUC, and then were incubated at 25°C for indicated time periods, separated by SDS-PAGE, transferred to PVDF membrane, and detected with the anti-Myc (Abmart, China).

### DNA and RNA Isolation and Real-Time PCR or RT-PCR Analysis

Total DNA was extracted from apical developing leaves using the DNAsecure Plant Kit (TIANGEN, China). Total RNA was extracted from apical developing leaves using the Trizol reagent (TIANGEN, China) and treated with RNase-free DNase I (Sigma-Aldrich). First strand cDNA was synthesized using 2–5 μg of total RNA with oligo-d(T) primer and M-MLV reverse transcriptase (TIANGEN, China). Real time RT-PCR was performed using Power SYBR Green PCR master mix (Life, USA). *EIF4a* and *Actin* were used as internal control for *N*. *benthamiana* for normalization. Primers were designed with Primer3web (http://primer3.ut.ee/) and listed in Supplemental Table S1. The values were calculated using the comparative normalized Ct method and all the experiments were repeated at least two times. Data were analyzed and plotted with Origin 8.1.

### Accession Number

Sequence data from this article can be found in the GenBank data libraries under accession numbers: *CLCuMuV* (GQ924756); *CLCuMuB* (GQ906588); *SlSKP1* (XM_004250675); *NbSKP1*.*1* (KP017273); *NbSKP1*.*2* (KP017274); *NbSKP1*.*3* (KP017275); *NbSKP1L1* (KP017276); *NbCUL1* (KP017277); *UBC3* (KR296788); *eIF4a* (KX247369); *Actin* (JQ256516); *PID* (KR082145); *COI1* (AF036340); *GAI* (KR082148); *GFP* (U87973); *Defensin-like protein 1* (KX139060); *Defensin-like protein 2* (KX139061); *Pathogen like protein* (KX139062); *Gibberellin- regulated protein 14* (KX139063); *Gibberellin-regulated protein 6* (KX139064); *SAUR14* (KX139065).

## Supporting Information

S1 Fig Schematic representation of the CLCuMuV, CLCuMuB βM1 and the βM2. The construct of CLCuMuV is a head-to-tail 1.7mer of CLCuMuV genome. The CLCuMuB consists of the *βC1* ORF, an A-rich region and the satellite conserved region (SCR). The stem-loop structure is shown. βM1 is a null mutant betasatellite for the βC1 gene with a ATG-TGA transition in the start codon. βM2 is a head-to-tail dimer of CLCuMuB genome with cloning sites of *Asc*I and *Xba*I in place of βC1 ORF. *NPTII* is a selective kanamycin resistance marker, *CaMV 35Sp* represents the *Cauliflower mosaic virus* 35S promoter. LB and RB stand for the left and right board of T-DNA. *Ubi3p* represents the *Solanum tuberosum ubiquitin-3* promoter. *ColE1* or PBR322 *ori* represents the plasmid replication origin in *E*.*coli*. *Rep oriV* or *PVS1 rep* represents the plasmid replication origin in *Agrobacterium*.
(TIF)

S2 Fig CLCuMuB βC1 enhances CLCuMuV accumulation and produces viral symptoms. (**A**) Healthy *N*. *benthamiana* and plants were infected by CLCuMuV with βM1 (CA+βM1) or CLCuMuB (CA+β). The photo was taken at 14 dpi. Different letters indicate significant differences (ANOVA, P < 0.05). (**B**) Total DNA was extracted from upper leaves of each plant respectively and subjected to quantitative real-time PCR to quantify viral DNA accumulation (means±SEM, n = 3). The internal reference method was used to calculate the relative amount of viral DNA.
(TIF)

S3 Fig The phenotype of CLCuMuB βC1 transgenic *N*. *benthamiana*. (**A and B**) Transgenic *N*. *benthamiana* lines that contain CLCuMuB βC1 gene under control of its own native promoter (*βC1pro*:*βC1*). (**C**) Transgenic *N*. *benthamiana* line that contains GFP-tagged CLCuMuB βC1 driven by CaMV 35S promoter (*35Spro*:*GFP-βC1*). (**D, E and F**) Transgenic *N*. *benthamiana* lines that contain HA-tagged CLCuMuB βC1 driven by CaMV 35S promoter (*35Spro*:*HA-βC1*). (**G**) Relative expression level of *βC1* in different lines of *βC1pro*:*βC1* (means±SEM, n = 3). *Actin* was used as the internal reference. (**H**) Relative protein level of GFP-βC1 in *35Spro*:*GFP-βC1*. (**I**) Relative protein level of HA-βC1 in different lines of *35Spro*:*HA-βC1*.
(TIF)

S4 Fig Amino acid sequence alignment of SKP1 proteins. Black, dark gray, light gray and white backgrounds represent residues that are conserved in 100%, above 80%, above 60%, below 60% of the sequences at the corresponding position respectively. Capital letters under each block indicate consensus residues that are conserved in all SKP1s and letters in lowercase indicate mostly conserved residues other than consensus ones. SKP1 were investigated as follows: AtASK1 (AT1G75950); AtASK2 (AT5G42190); SlSKP1 (XM_004250675); NbSKP1.1 (KP017273); NbSKP1.2 (KP017274); NbSKP1.3 (KP017275); NbSKP1L1 (KP017276).
(TIF)

S5 Fig The reverse Co-IP of CLCuMuB βC1 with NbSKP1 and protein level of BiFC. (**A**) Reverse co-immunoprecipitation (co-IP) assays show that CLCuMuB βC1 interacted with NbSKP1.1 and NbSKP1L1 *in vivo*. GUS tagged with HA (HA-GUS), HA-NbSKP1.1 or HA-NbSKP1L1 was co-expressed with GFP-βC1 in *N*. *benthamiana* leaves by agroinfiltration. At 48 hpi, leaf lysates were immunoprecipitated (IP) with HA agarose (Abmart, China), then the immunopercipitates were detected by western blotting (IB) using anti-GFP and anti-HA antibodies. (**B**) All plasmids used in BiFC assays can be expressed correctly. Leaf samples were grinded by liquid nitrogen and added 2×loading buffer (100 mg: 200 μL). After 100°C for 10 min, protein samples were used to do western blot assays by the anti-HA antibody. The PVDF membrane was stained with Ponceaux to visualize the large subunit of ribulose-1,5-bisphosphate as a loading control.
(TIF)

S6 Fig βC1ΔC43 does not produce viral symptoms. (**A**) Six- to seven-week-old *N*. *benthamiana* plants were agroinoculated with PVX-cLUC (Control), PVX-βC1 and PVX-βC1ΔC43. Phenotype of plants at 14 dpi was shown. (**B**) Real-time results show relative expression level (means±SEM, n = 3) of *βC1* and *βC1ΔC43* at 14 dpi. *Actin* was used as internal references.
(TIF)

S7 Fig CLCuMuB-based silencing of *PDS* mainly occurs in vascular tissues. Six- to seven-week-old *N*. *benthamiana* plants were agroinoculated with CLCuMuV and βM2-*PDS* at 25 dpi.
(TIF)

S8 Fig The position relationship of different VIGS fragments for *NbSKP1*.*1*, *NbCUL1* and *NbUBC3*. The position relationship among 176-bp, 184-bp and 345-bp *NbSKP1*.*1* fragments, 268-bp and 345-bp *NbCUL1* fragments and the 345-bp *NbUBC3* fragment for silencing were shown.
(TIF)

S9 Fig Relative expression level of *NbSKP1s* and *NbSKP1L1* in *N*. *benthamiana*. Total RNA of healthy *N*. *benthamiana* was subjected to quantitative real-time RT-PCR to quantify the expression level of *NbSKP1s* and *NbSKP1L1* (means±SEM, n = 3). *EIF4a* was used as the internal reference. These experiments were repeated twice.
(TIF)

S10 Fig Silencing of *NbSKP1*s leads to growth retardation and severe viral symptoms of *N*. *benthemiana*. Silencing of *NbSKP1s* via CLCuMuV (CA) and βM2-*SKP1*F3 led growth retardation symptoms to emerge in partial infected plants at 45 dpi.
(TIF)

S11 Fig Using βM2-*GFP*F as the control gets similar results. (**A**) Six- to seven-week-old *N*. *benthamiana* plants were agroinoculated with CLCuMuV (CA) and βM2-*GFP*F (as the control) or βM2-*SKP1*F3. (**B**) Silencing of *NbSKP1s* enhanced CLCuMuV DNA accumulation. 7 plants for each group. At 14 dpi, total DNA was extracted from upper leaves of each plant respectively and subjected to quantitative real-time PCR (means±SEM, n = 7) to quantify viral DNA accumulation. The internal reference method was used to calculate the relative amount of viral DNA. (**C**) Severe symptoms of plants infected with CLCuMuV and βM2-*SKP1*F3 at 21 dpi. (**D**) Real-time RT-PCR confirmed silencing of *NbSKP1s*. Total RNA was extracted from each plant respectively and subjected to quantitative RT-PCR (means±SEM, n = 3) to quantify *NbSKP1s* mRNA level. *Actin* was used as the internal reference. The raw data of (B) and (D) were analysed by two-sample ***t***-test to show the significance level at 0.05 (*), 0.01 (**) or 0.001(***). These experiments were repeated at least twice.
(TIF)

S12 Fig Silencing *NbSKP1s* via TYLCCNB-based VIGS system enhances CLCuMuV DNA accumulation and results in typical viral symptoms. (**A**) Six- to seven-week-old *N*. *benthamiana* plants were agroinoculated with CLCuMuV (CA), TYLCCNV (TA) and 2mβ-*GFP*F1 (as the control) or 2mβ-*SKP1*F3. (**B**) Silencing of *NbSKP1s* enhanced CLCuMuV DNA accumulation. 7 plants for each group. At 14 dpi, total DNA was extracted from upper leaves of each plant respectively and subjected to quantitative real-time PCR (means±SEM, n = 7) to quantify viral DNA accumulation. The internal reference method was used to calculate the relative amount of viral DNA. (**C**) Severe symptoms of all plants infected with CA, TA and 2mβ-*SKP1*F3 at 21 dpi. (**D**) Real-time RT-PCR confirmed silencing of *NbSKP1s*. Total RNA was extracted from each plant respectively and subjected to quantitative RT-PCR (means±SEM, n = 3) to quantify *NbSKP1s* mRNA level. *Actin* was used as the internal reference. The raw data of (B) and (D) were analysed by two-sample ***t***-test to show the significance level at 0.05 (*). These experiments were repeated at least twice.
(TIF)

S13 Fig CLCuMuB βC1 reduced auxin and GA response in transgenic *N*. *benthemiana* lines. (**A**) Relative expression level of marker genes of gibberellins response in HA-βC1 transgenic (#2 HA-βC1 and #3 HA-βC1) and wild-type *N*. *benthamiana* (#2 Control and #3 Control) seedlings determined by quantitative real-time PCR. #2 HA-βC1 and #2 WT were presented on same plates, while #3 HA-βC1 and #3 WT were presented on same plates. HA-βC1-expressing lines are compared with their corresponding control. (**B**) Relative expression level of marker genes of gibberellins response in HA-βC1 transgenic and wild-type *N*. *benthamiana* (Control) seedlings determined by quantitative real-time PCR. *Actin* was used as the internal reference. Bars represent SEM. The raw data were analysed by two-sample ***t***-test to show the significance level at 0.05 (*), 0.01 (**) and 0.001 (***). These experiments were repeated at least twice.
(TIF)

S14 Fig CLCuMuB βC1 does not repress JA biosynthesis. JA levels in healthy *N*.*benthamiana* plants or plants infected by CA+β and CA+βM1. Different letters indicate significant differences (ANOVA, P < 0.05).
(TIF)

S15 Fig Myc-COI1 interacts with SCF complexes *in vivo*. Co-immunoprecipitation (co-IP) assays show that Myc-COI1 interacted with NbSKP1.1 and NbCUL1 *in vivo*. GFP-CUL1 or GFP (as a negative control) was co-expressed with HA-NbSKP1.1 and Myc-COI1 in *N*.*benthamiana* leaves by agroinfiltration. At 48 hpi, leaf lysates were immunoprecipitated (IP) with GFP-Trap agarose, then the immunopercipitates were detected by western blotting using anti-GFP, anti-HA and anti-Myc antibodies.
(TIF)

S16 Fig Transgenic expression of βC1 reduces accumulation of COI1 *in vivo*. GFP (as the control) or Myc-COI1 was agroinoculated into eight- to nine-week-old wild-type (WT) or HA-βC1 transgenic *N*. *benthamiana* plants (#2 and #3). At 48 hpi, leaf lysates were analysed by western blot via anti-Myc or anti-GFP antibody. Intensity was detected through Total Lab TL120. Relative mRNA levels of *GFP* and *Myc-COI1* were quantified via real-time PCR. To exclude influence from endogenous COI, 5’UTR and Myc tag sequences were used to design primers. *Actin* was used as the internal reference. These experiments were repeated three times.
(TIF)

S17 Fig CLCuMuB βC1ΔC43 doesn’t hinder the degradation of YFP-GAI *in vivo*. (**A**) CLCuMuB βC1 attenued degradation of YFP-GAI *in vivo*. YFP-GAI expression construct was coinfiltrated with constructs expressing HA-βC1ΔC43 or HA-βC1 into seven to eight-week-old *N*. *benthamiana* plant leaves. Around 48 hpi, agroinfiltrated leaves were sprayed with 100 μM GA_3_ or mock solution (ethonal) and visualized via a Zeiss LSM 710 laser scanning microscope. Bar scale represents 200 μm. DMSO and MG132 (50 μM) were applied into plant leaves 12 h before observation. Protein samples were used to do SDS-PAGE and western blot analysis with the anti-GFP antibody, which also recognizes YFP. The PVDF membrane was stained with Ponceaux to visualize the large subunit of ribulose-1,5-bisphosphate as a loading control. (**B**) Real-time RT-PCR detected the mRNA level of YFP-GAI. Total RNA was extracted from each *N*. *benthamiana* leaves and then subjected to quantitative RT-PCR (means±SEM, n = 3) to quantify YFP-GAI mRNA level. *Actin* was used as the internal reference. (**C**) CLCuMB βC1 didn’t affect stability of GFP *in vivo*. Detection of GFP (as an internal control) in *N*. *benthamiana* leaves coinfiltrated with the construct expressing GFP together with constructs expressing HA-βC1ΔC43 or HA-βC1 and treated with 100 μM GA_3_ or mock (ethanol) solution and visualized via a Zeiss LSM 710 laser scanning microscope. Bar scale represents 200 μm. Protein samples were subjected to SDS-PAGE and immunoblot analysis with anti-GFP. The PVDF membrane was stained with Ponceaux to visualize the large subunit of ribulose-1,5-bisphosphate as a loading control.
(TIF)

S18 Fig Silencing of *UBC3* does not lead to typical viral symptoms and increased CLCuMuV DNA accumulation. (A) Six- to seven-week-old *N*. *benthamiana* plants were agroinoculated with CLCuMuV and βM2-*UBC3*F which is resulted by introducing a 345-bp fragment of *UBC3* into βM2. (B) Silencing of *UBC3* led to no enhancement on virus accumulation. 7 plants for each group. At 14 dpi, total DNA was extracted from upper leaves of each plant respectively and subjected to quantitative real-time PCR (means±SEM, n = 7) to quantify viral DNA accumulation. The internal reference method was used to calculate the relative amount of viral DNA. (C) Silencing of *UBC3* led to no typical symptom even at 21 dpi. (D) Real-time RT-PCR confirmed silencing of *NbSKP1s*. Total RNA was extracted from each plant respectively and subjected to quantitative RT-PCR (means±SEM, n = 4) to quantify *UBC3* mRNA level. *Actin* was used as the internal reference. The raw data of (B) and (D) were analysed by two-sample *t*-test to show the significance level at 0.05 (*). These experiments were repeated at least twice.
(TIF)

S19 Fig Silencing of *NbSKP1s* via CA+βM2-*SKP1*-176 enhances virus accumulation but leads no typical viral symptoms. (**A1, A2 and A3**) Six- to seven-week-old *N*. *benthamiana* plants were agroinoculated with CLCuMuV (CA) and βM2-*SKP1*-176 (A1), βM2-*SKP1*-184 (A2), βM2-*SKP1*-351 (A3) or βM2 (as the control). (**B1, B2 and B3**) Silencing of *NbSKP1s* enhanced CLCuMuV DNA accumulation. At 14 dpi, total DNA was extracted from each plant respectively and subjected to quantitative real-time PCR (means±SEM, n ≥7) to quantify viral DNA accumulation. *EIF4a* was used as the internal reference to calculate the relative amount of viral DNA. (**C1, C2 and C3**) Real-time RT-PCR confirmed silencing of *NbSKP1s*. Total RNA was extracted from upper leaves of each plant respectively and subjected to quantitative RT-PCR (means±SEM, n = 4) to quantify *NbSKP1s* mRNA level. *EIF4a* was used as the internal reference. The raw data of (B1–B3) and (C1–C3) were analysed by two-sample ***t***-test to show the significance level at 0.05 (*), 0.01 (**) and 0.001(***). These experiments were repeated at least twice. (**D1, D2 and D3**) Symptoms of plants infected with CLCuMuV (CA) and βM2-*SKP1*-176 (A1), βM2-*SKP1*-184 (A2) or βM2-*SKP1*-351 (A3) at 21 dpi. No plants infected with CA+βM2-*SKP1*-176, about 50% plants infected with CA+βM2-*SKP1*-184 and all plants infected with CA+βM2-*SKP1*-351 showed typical symptoms.
(TIF)

S1 Table Primers used in vector construction and PCR analysis. (PDF)
