# Supplementary figures and images for: CLCuMuB βC1 Subverts Ubiquitination by Interacting with NbSKP1s to Enhance Geminivirus Infection in Nicotiana benthamiana
Source: PLoS Pathog. 2016 Jun 17;12(6):e1005668. doi: 10.1371/journal.ppat.1005668 (PMC4912122; doi:10.1371/journal.ppat.1005668)

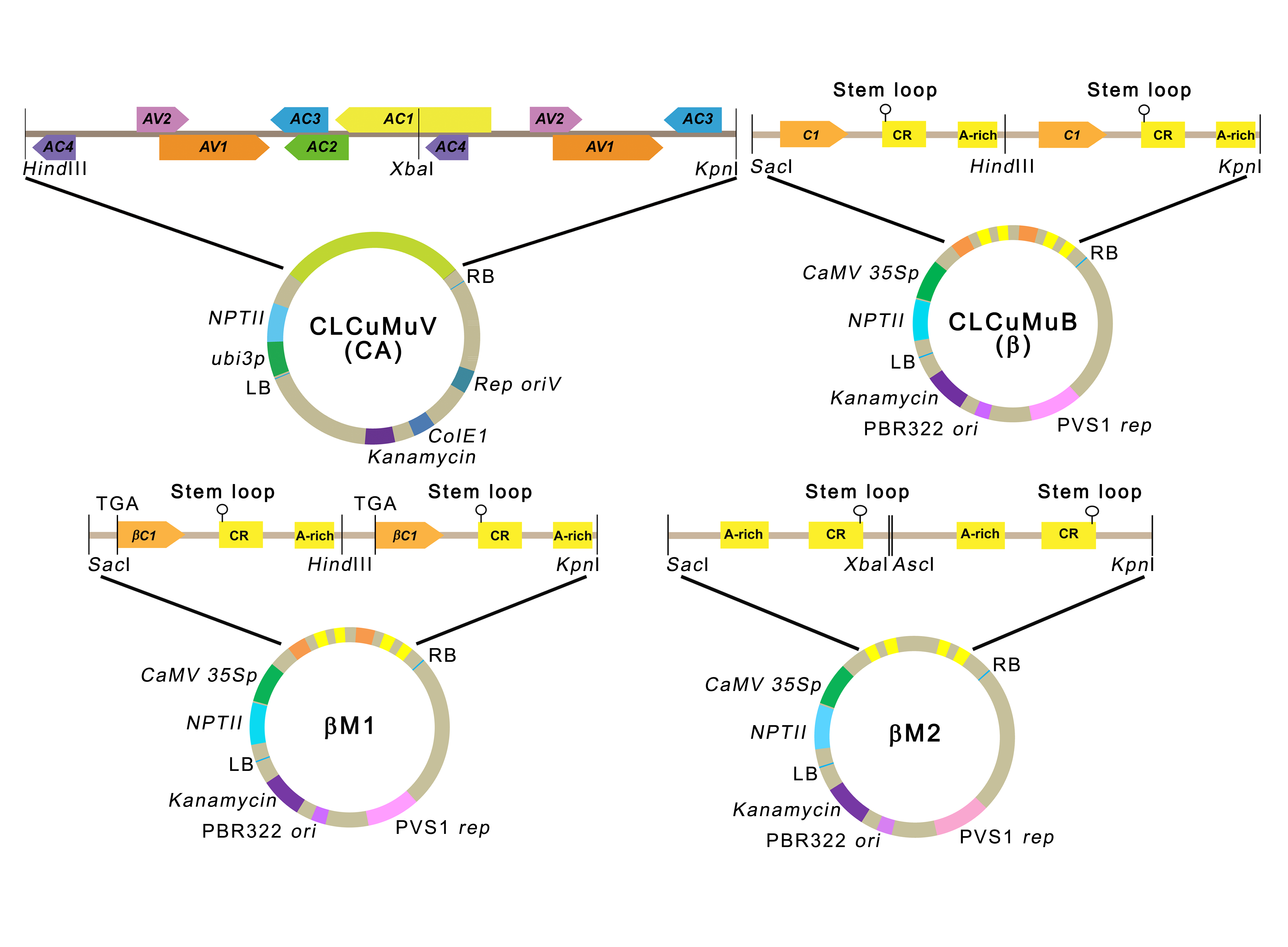

Supplement: S1 Fig — The construct of CLCuMuV is a head-to-tail 1.7mer of CLCuMuV genome. The CLCuMuB consists of the βC1 ORF, an A-rich region and the satellite conserved region (SCR). The stem-loop structure is shown. βM1 is a null mutant betasatellite for the βC1 gene with a ATG-TGA transition in the start codon. βM2 is a head-to-tail dimer of CLCuMuB genome with cloning sites of AscI and XbaI in place of βC1 ORF. NPTII is a selective kanamycin resistance marker, CaMV 35Sp represents the Cauliflower mosaic virus 35S promoter. LB and RB stand for the left and right board of T-DNA. Ubi3p represents the Solanum tuberosum ubiquitin-3 promoter. ColE1 or PBR322 ori represents the plasmid replication origin in E.coli. Rep oriV or PVS1 rep represents the plasmid replication origin in Agrobacterium. (TIF) [file ppat.1005668.s001.tif]

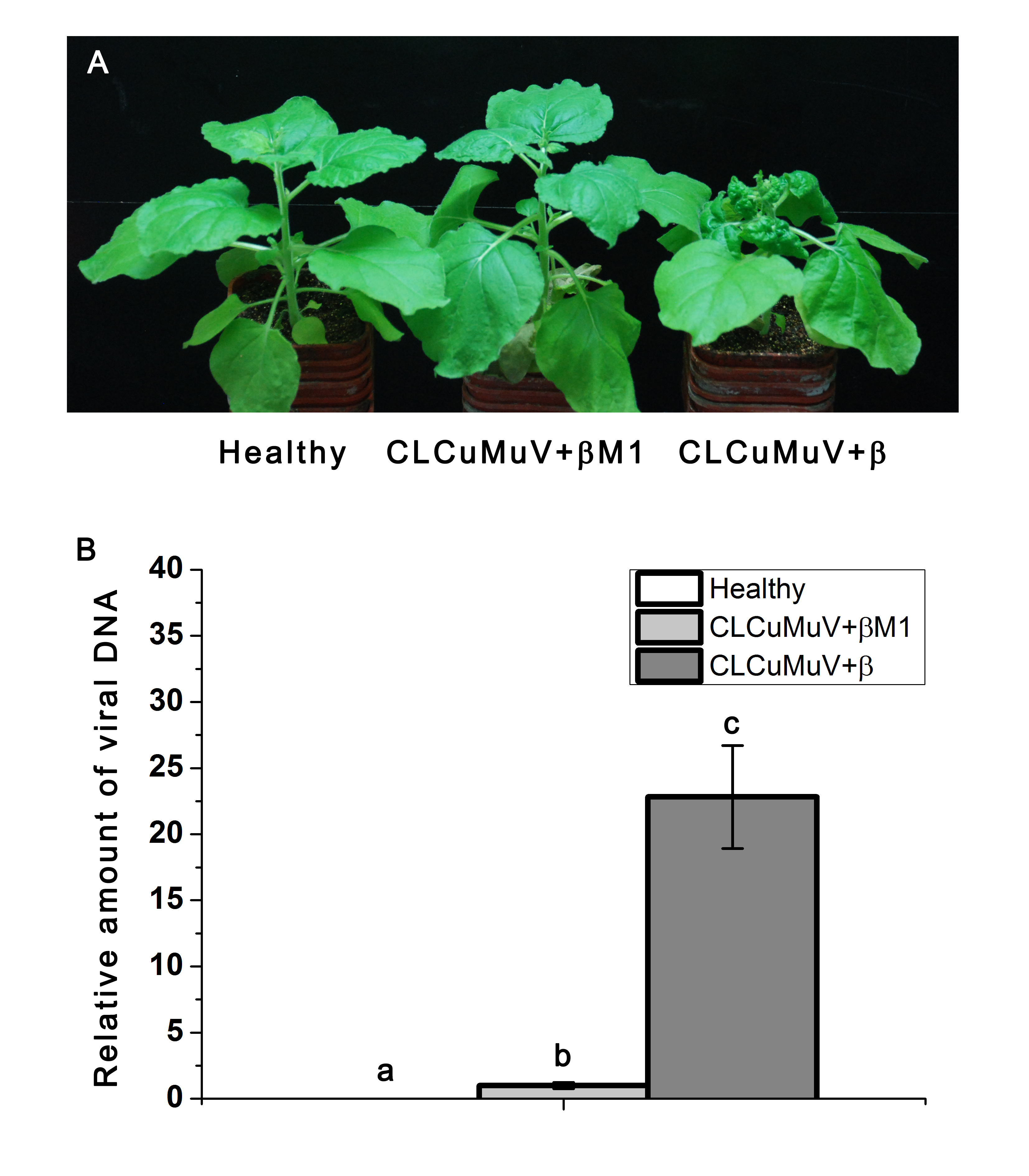

Supplement: S2 Fig — (A) Healthy N. benthamiana and plants were infected by CLCuMuV with βM1 (CA+βM1) or CLCuMuB (CA+β). The photo was taken at 14 dpi. Different letters indicate significant differences (ANOVA, P < 0.05). (B) Total DNA was extracted from upper leaves of each plant respectively and subjected to quantitative real-time PCR to quantify viral DNA accumulation (means±SEM, n = 3). The internal reference method was used to calculate the relative amount of viral DNA. (TIF) [file ppat.1005668.s002.tif]

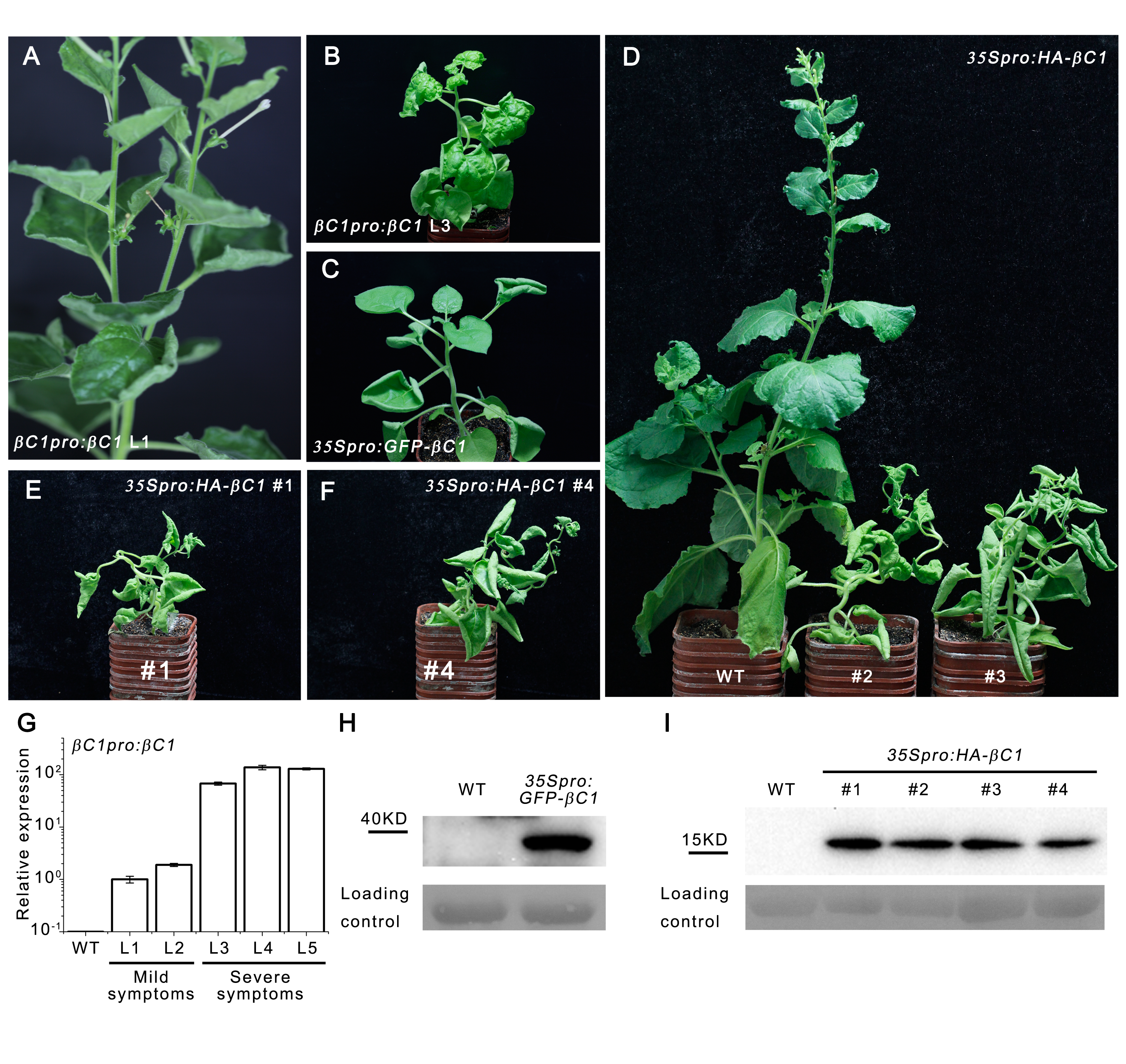

Supplement: S3 Fig — (A and B) Transgenic N. benthamiana lines that contain CLCuMuB βC1 gene under control of its own native promoter (βC1pro:βC1). (C) Transgenic N. benthamiana line that contains GFP-tagged CLCuMuB βC1 driven by CaMV 35S promoter (35Spro:GFP-βC1). (D, E and F) Transgenic N. benthamiana lines that contain HA-tagged CLCuMuB βC1 driven by CaMV 35S promoter (35Spro:HA-βC1). (G) Relative expression level of βC1 in different lines of βC1pro:βC1 (means±SEM, n = 3). Actin was used as the internal reference. (H) Relative protein level of GFP-βC1 in 35Spro:GFP-βC1. (I) Relative protein level of HA-βC1 in different lines of 35Spro:HA-βC1. (TIF) [file ppat.1005668.s003.tif]

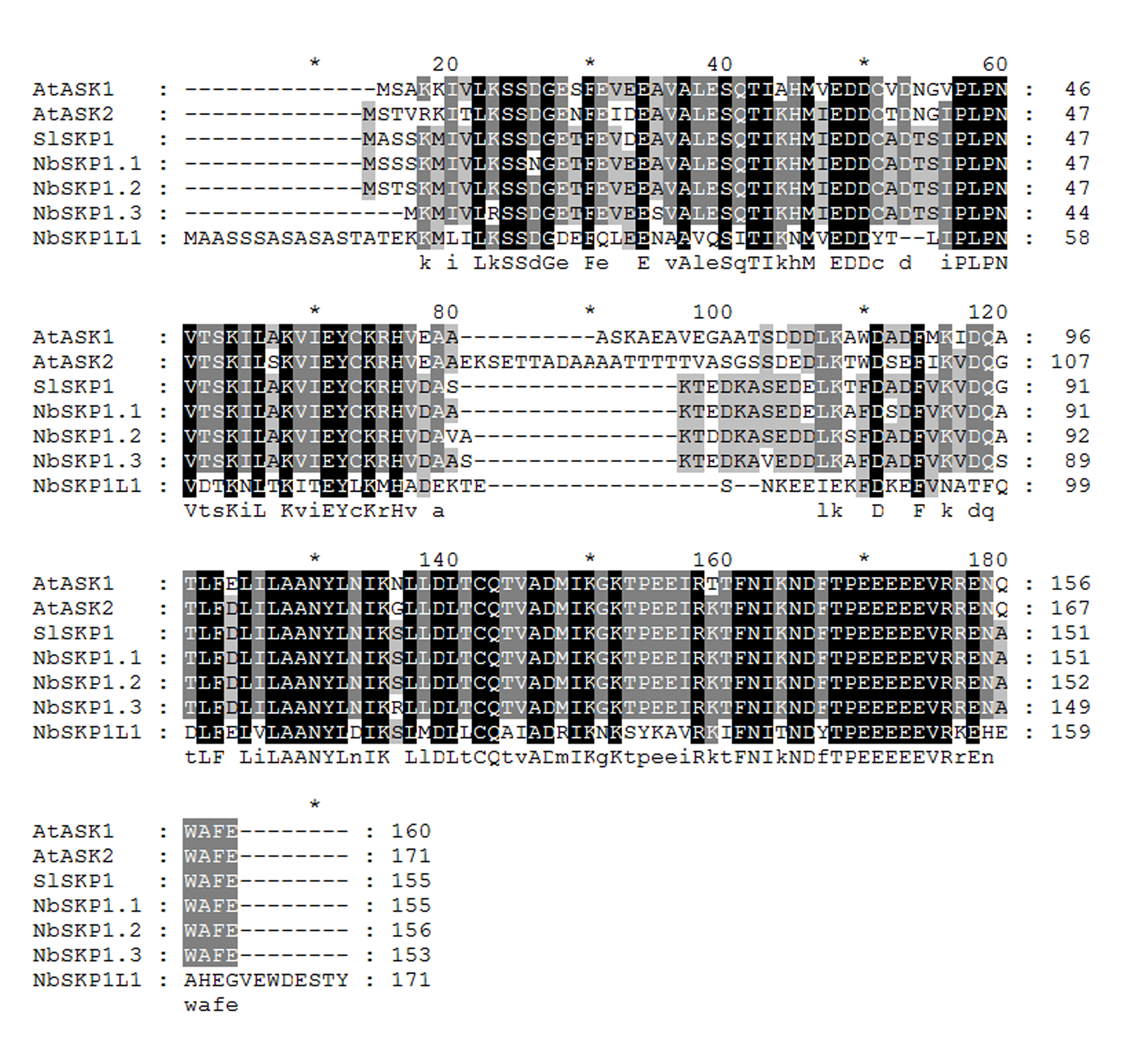

Supplement: S4 Fig — Black, dark gray, light gray and white backgrounds represent residues that are conserved in 100%, above 80%, above 60%, below 60% of the sequences at the corresponding position respectively. Capital letters under each block indicate consensus residues that are conserved in all SKP1s and letters in lowercase indicate mostly conserved residues other than consensus ones. SKP1 were investigated as follows: AtASK1 (AT1G75950); AtASK2 (AT5G42190); SlSKP1 (XM_004250675); NbSKP1.1 (KP017273); NbSKP1.2 (KP017274); NbSKP1.3 (KP017275); NbSKP1L1 (KP017276). (TIF) [file ppat.1005668.s004.tif]

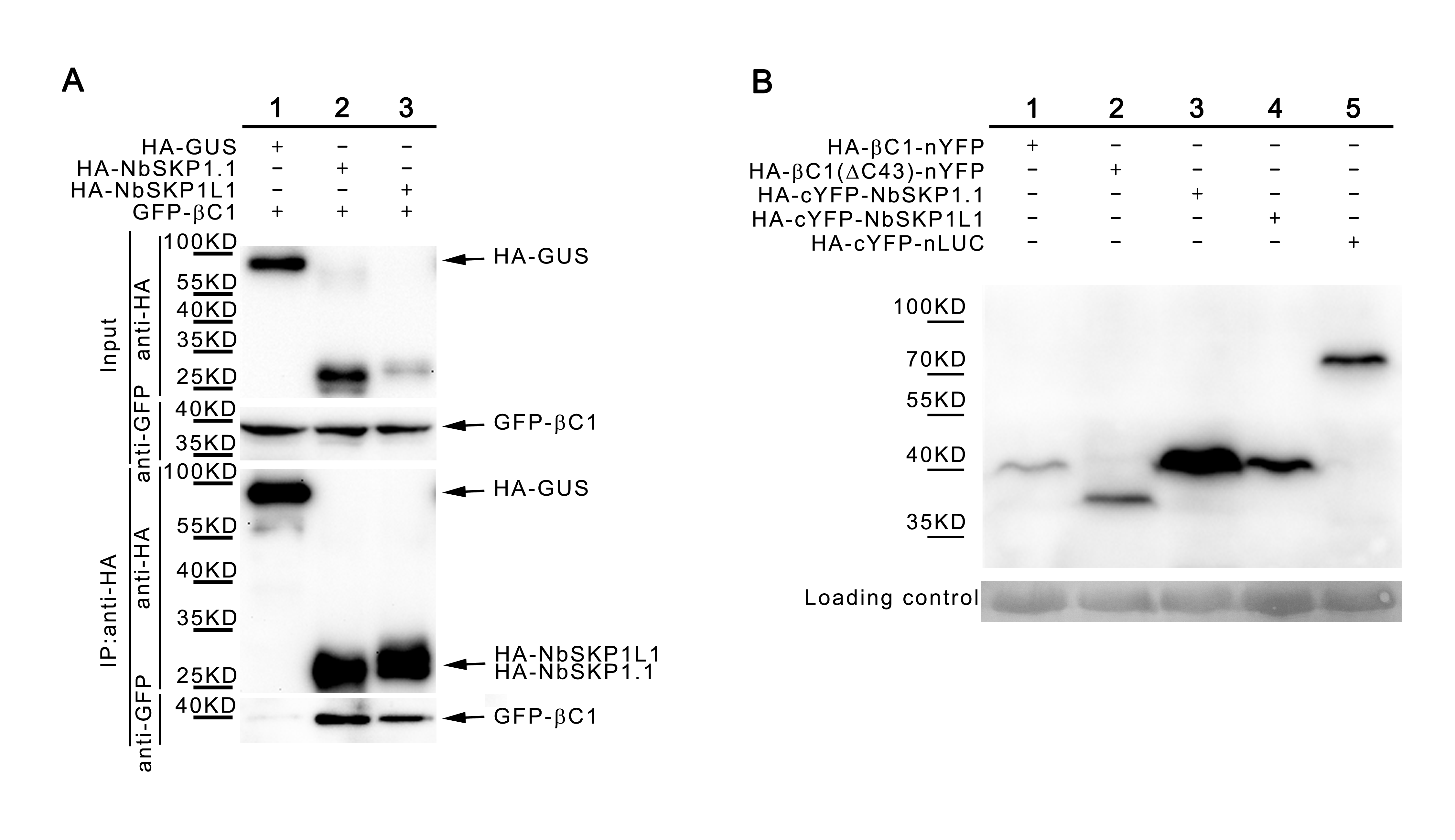

Supplement: S5 Fig — (A) Reverse co-immunoprecipitation (co-IP) assays show that CLCuMuB βC1 interacted with NbSKP1.1 and NbSKP1L1 in vivo. GUS tagged with HA (HA-GUS), HA-NbSKP1.1 or HA-NbSKP1L1 was co-expressed with GFP-βC1 in N. benthamiana leaves by agroinfiltration. At 48 hpi, leaf lysates were immunoprecipitated (IP) with HA agarose (Abmart, China), then the immunopercipitates were detected by western blotting (IB) using anti-GFP and anti-HA antibodies. (B) All plasmids used in BiFC assays can be expressed correctly. Leaf samples were grinded by liquid nitrogen and added 2×loading buffer (100 mg: 200 μL). After 100°C for 10 min, protein samples were used to do western blot assays by the anti-HA antibody. The PVDF membrane was stained with Ponceaux to visualize the large subunit of ribulose-1,5-bisphosphate as a loading control. (TIF) [file ppat.1005668.s005.tif]

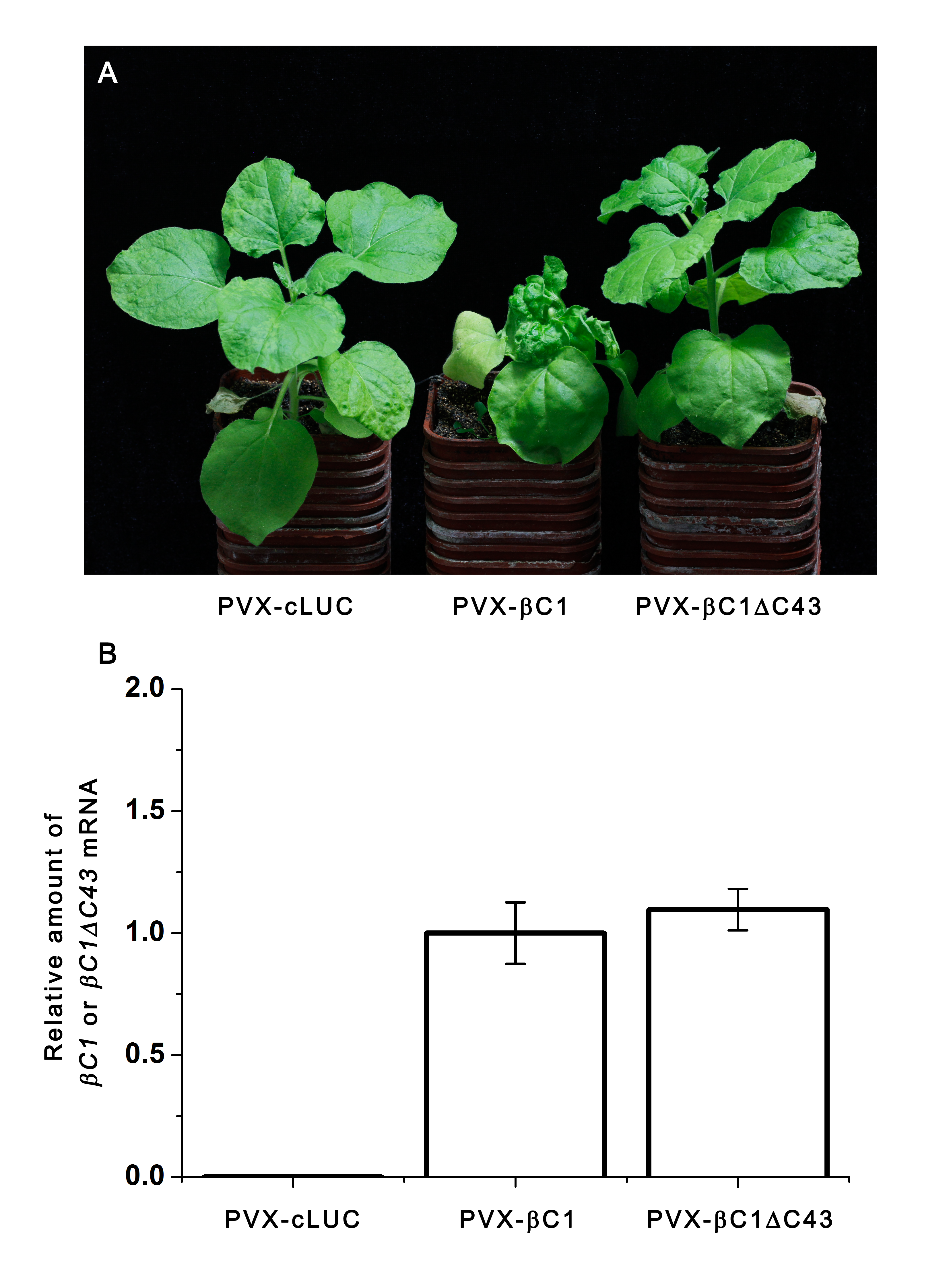

Supplement: S6 Fig — (A) Six- to seven-week-old N. benthamiana plants were agroinoculated with PVX-cLUC (Control), PVX-βC1 and PVX-βC1ΔC43. Phenotype of plants at 14 dpi was shown. (B) Real-time results show relative expression level (means±SEM, n = 3) of βC1 and βC1ΔC43 at 14 dpi. Actin was used as internal references. (TIF) [file ppat.1005668.s006.tif]

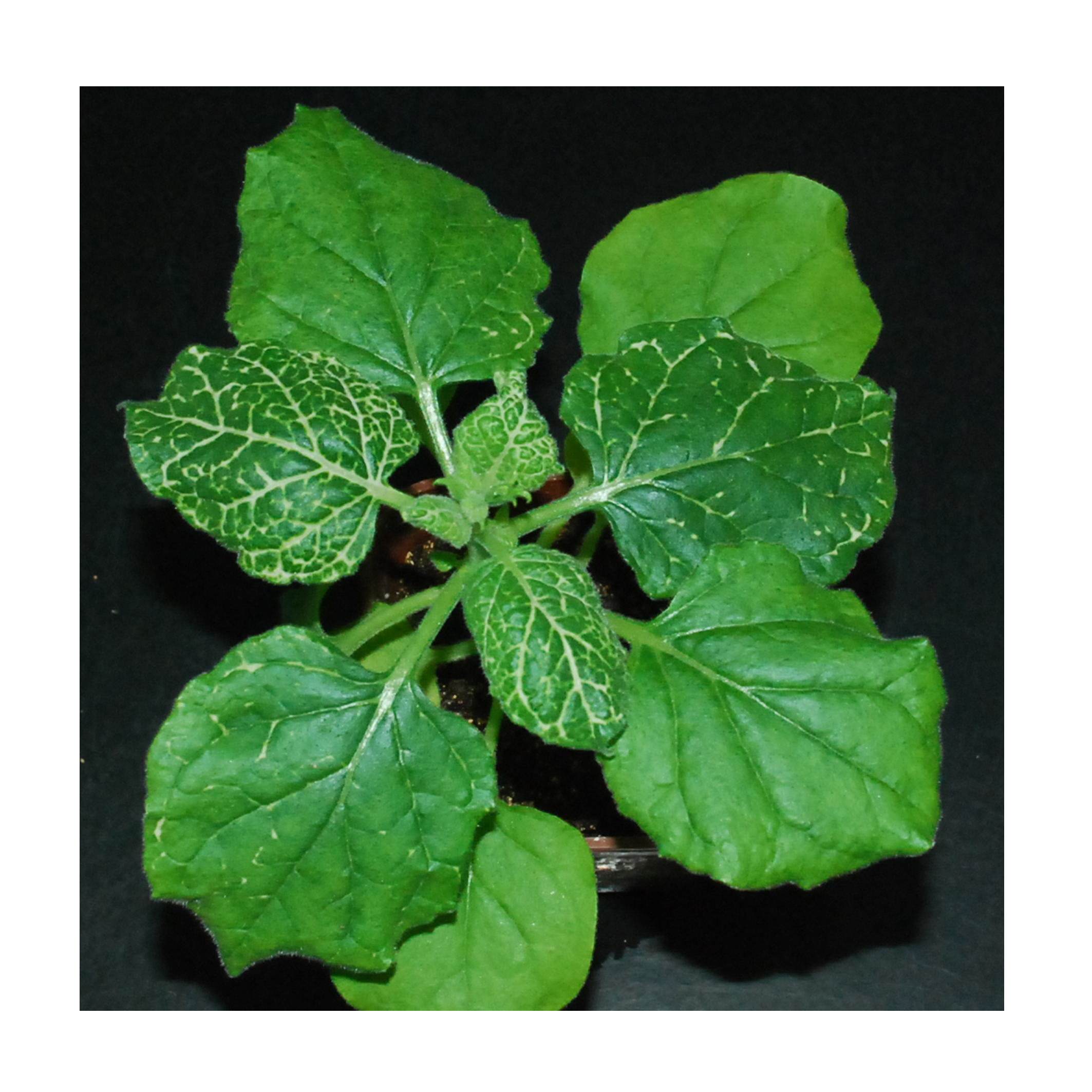

Supplement: S7 Fig — Six- to seven-week-old N. benthamiana plants were agroinoculated with CLCuMuV and βM2-PDS at 25 dpi. (TIF) [file ppat.1005668.s007.tif]

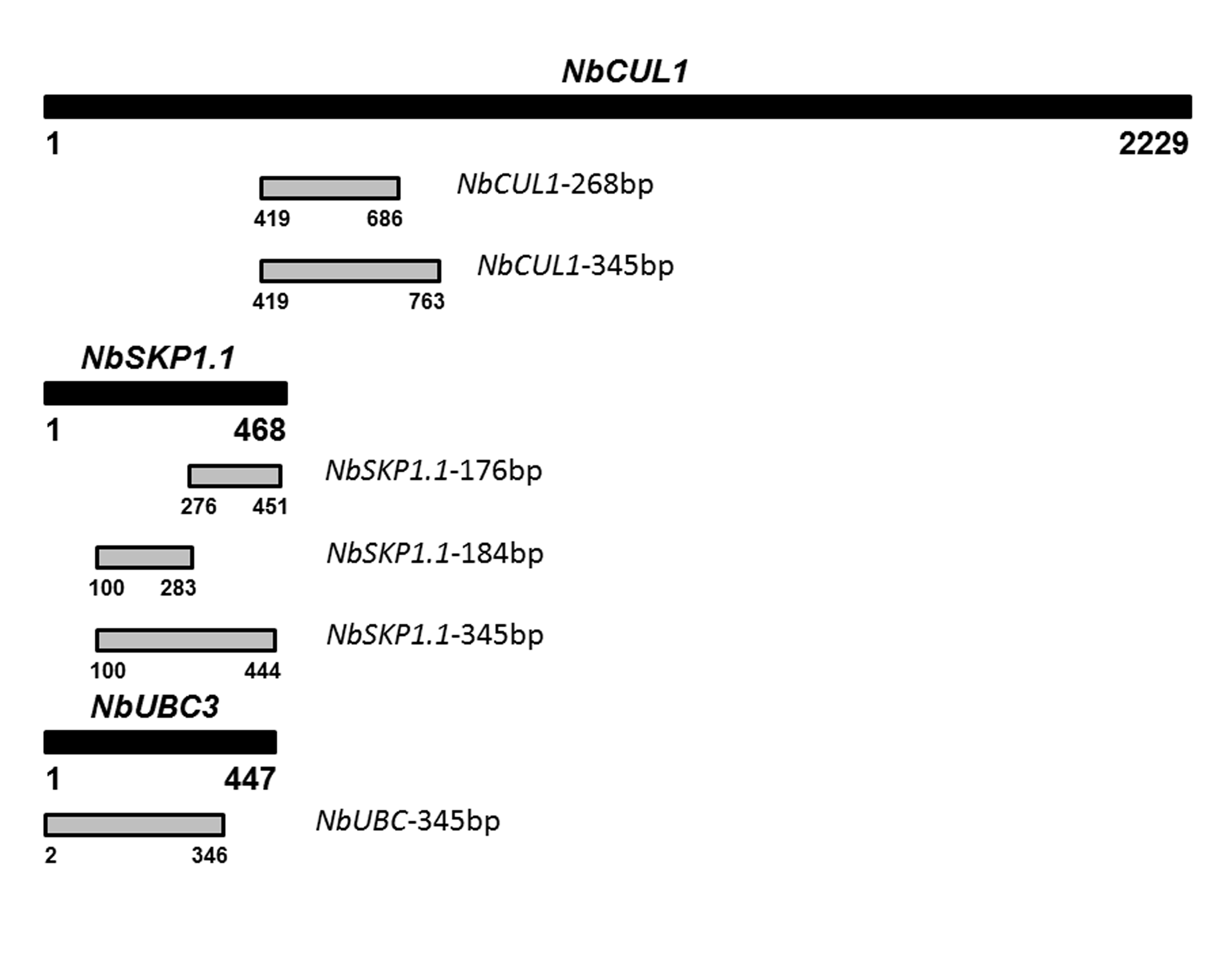

Supplement: S8 Fig — The position relationship among 176-bp, 184-bp and 345-bp NbSKP1.1 fragments, 268-bp and 345-bp NbCUL1 fragments and the 345-bp NbUBC3 fragment for silencing were shown. (TIF) [file ppat.1005668.s008.tif]

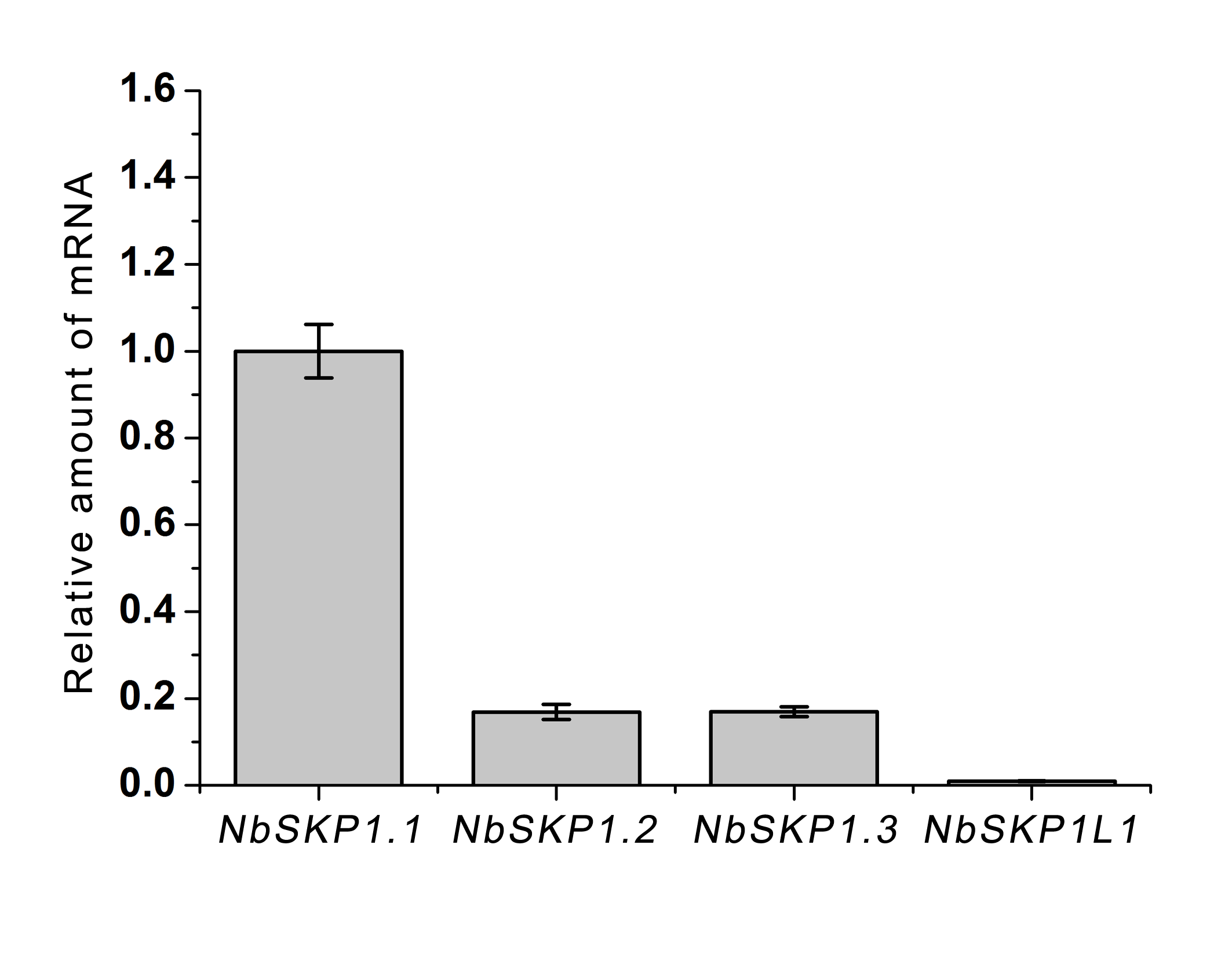

Supplement: S9 Fig — Total RNA of healthy N. benthamiana was subjected to quantitative real-time RT-PCR to quantify the expression level of NbSKP1s and NbSKP1L1 (means±SEM, n = 3). EIF4a was used as the internal reference. These experiments were repeated twice. (TIF) [file ppat.1005668.s009.tif]

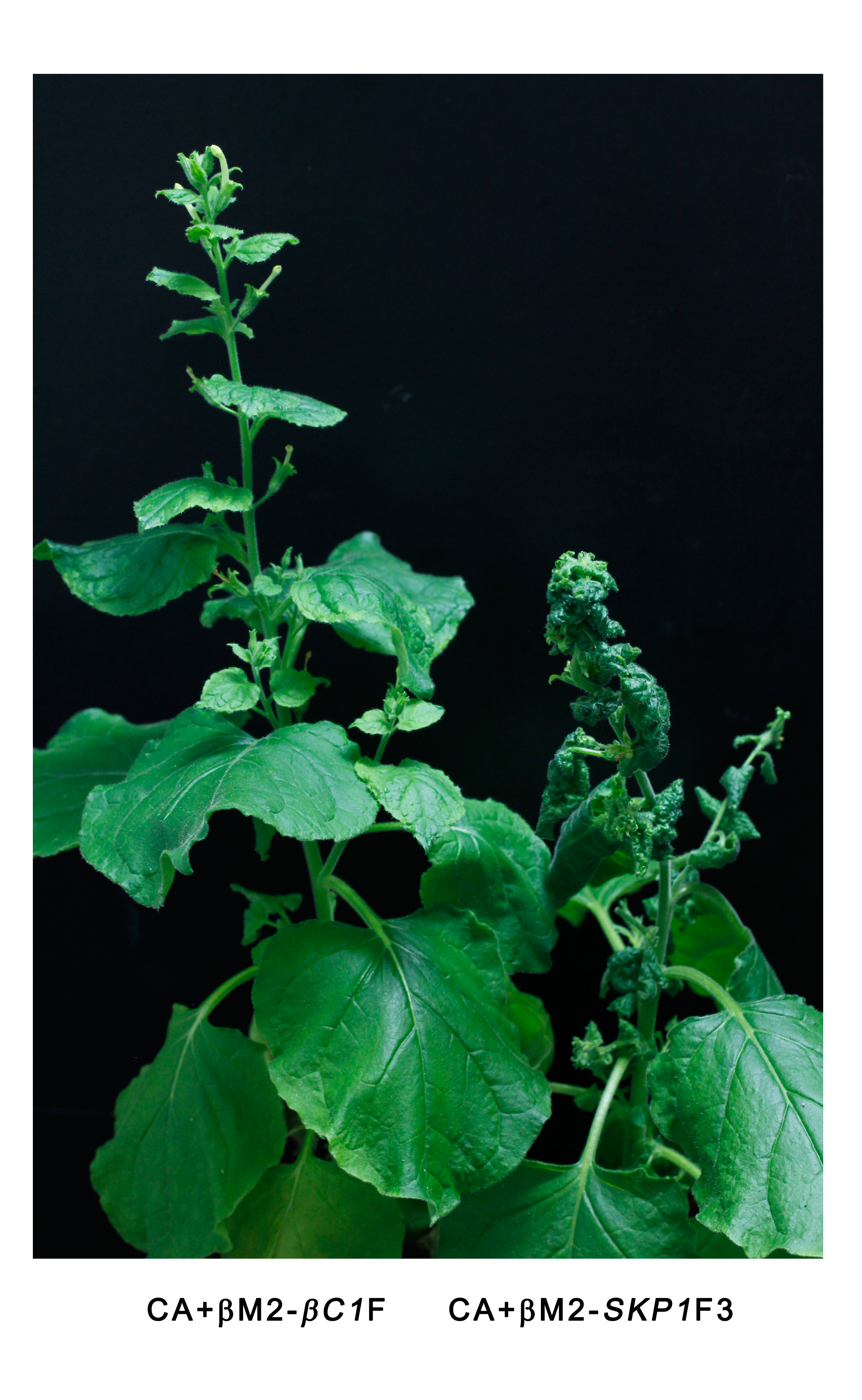

Supplement: S10 Fig — Silencing of NbSKP1s via CLCuMuV (CA) and βM2-SKP1F3 led growth retardation symptoms to emerge in partial infected plants at 45 dpi. (TIF) [file ppat.1005668.s010.tif]

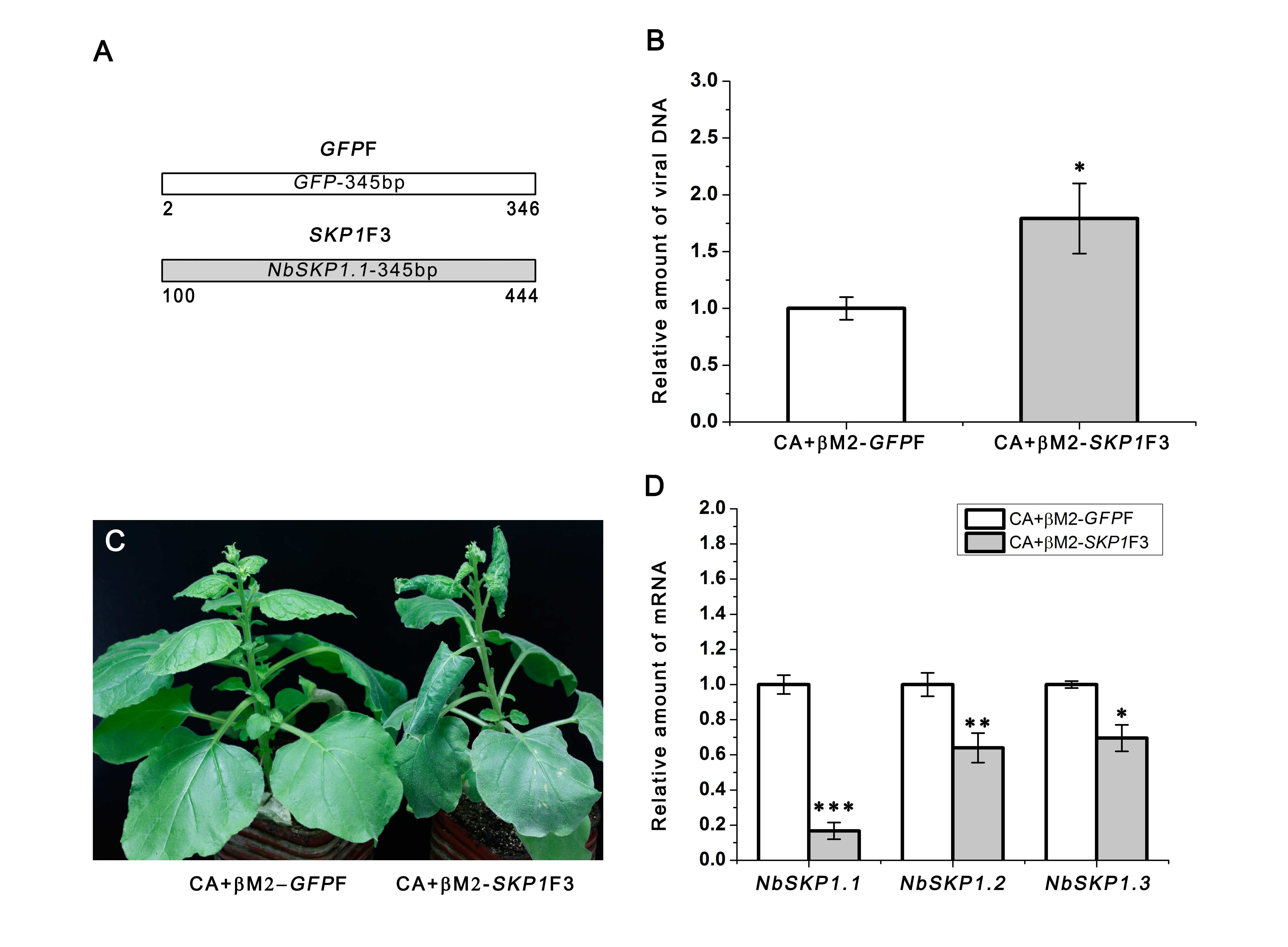

Supplement: S11 Fig — (A) Six- to seven-week-old N. benthamiana plants were agroinoculated with CLCuMuV (CA) and βM2-GFPF (as the control) or βM2-SKP1F3. (B) Silencing of NbSKP1s enhanced CLCuMuV DNA accumulation. 7 plants for each group. At 14 dpi, total DNA was extracted from upper leaves of each plant respectively and subjected to quantitative real-time PCR (means±SEM, n = 7) to quantify viral DNA accumulation. The internal reference method was used to calculate the relative amount of viral DNA. (C) Severe symptoms of plants infected with CLCuMuV and βM2-SKP1F3 at 21 dpi. (D) Real-time RT-PCR confirmed silencing of NbSKP1s. Total RNA was extracted from each plant respectively and subjected to quantitative RT-PCR (means±SEM, n = 3) to quantify NbSKP1s mRNA level. Actin was used as the internal reference. The raw data of (B) and (D) were analysed by two-sample t-test to show the significance level at 0.05 (*), 0.01 (**) or 0.001(***). These experiments were repeated at least twice. (TIF) [file ppat.1005668.s011.tif]

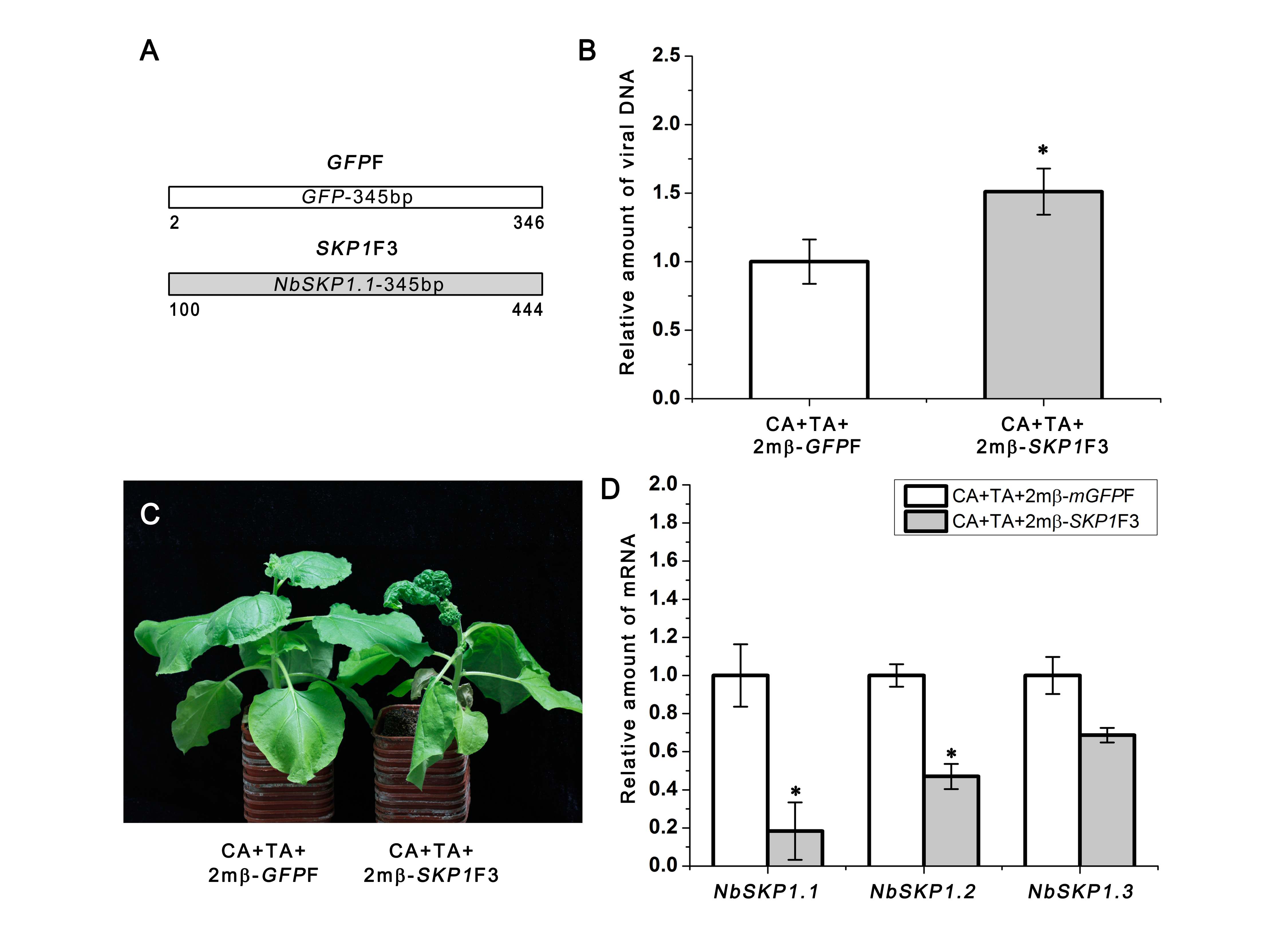

Supplement: S12 Fig — (A) Six- to seven-week-old N. benthamiana plants were agroinoculated with CLCuMuV (CA), TYLCCNV (TA) and 2mβ-GFPF1 (as the control) or 2mβ-SKP1F3. (B) Silencing of NbSKP1s enhanced CLCuMuV DNA accumulation. 7 plants for each group. At 14 dpi, total DNA was extracted from upper leaves of each plant respectively and subjected to quantitative real-time PCR (means±SEM, n = 7) to quantify viral DNA accumulation. The internal reference method was used to calculate the relative amount of viral DNA. (C) Severe symptoms of all plants infected with CA, TA and 2mβ-SKP1F3 at 21 dpi. (D) Real-time RT-PCR confirmed silencing of NbSKP1s. Total RNA was extracted from each plant respectively and subjected to quantitative RT-PCR (means±SEM, n = 3) to quantify NbSKP1s mRNA level. Actin was used as the internal reference. The raw data of (B) and (D) were analysed by two-sample t-test to show the significance level at 0.05 (*). These experiments were repeated at least twice. (TIF) [file ppat.1005668.s012.tif]

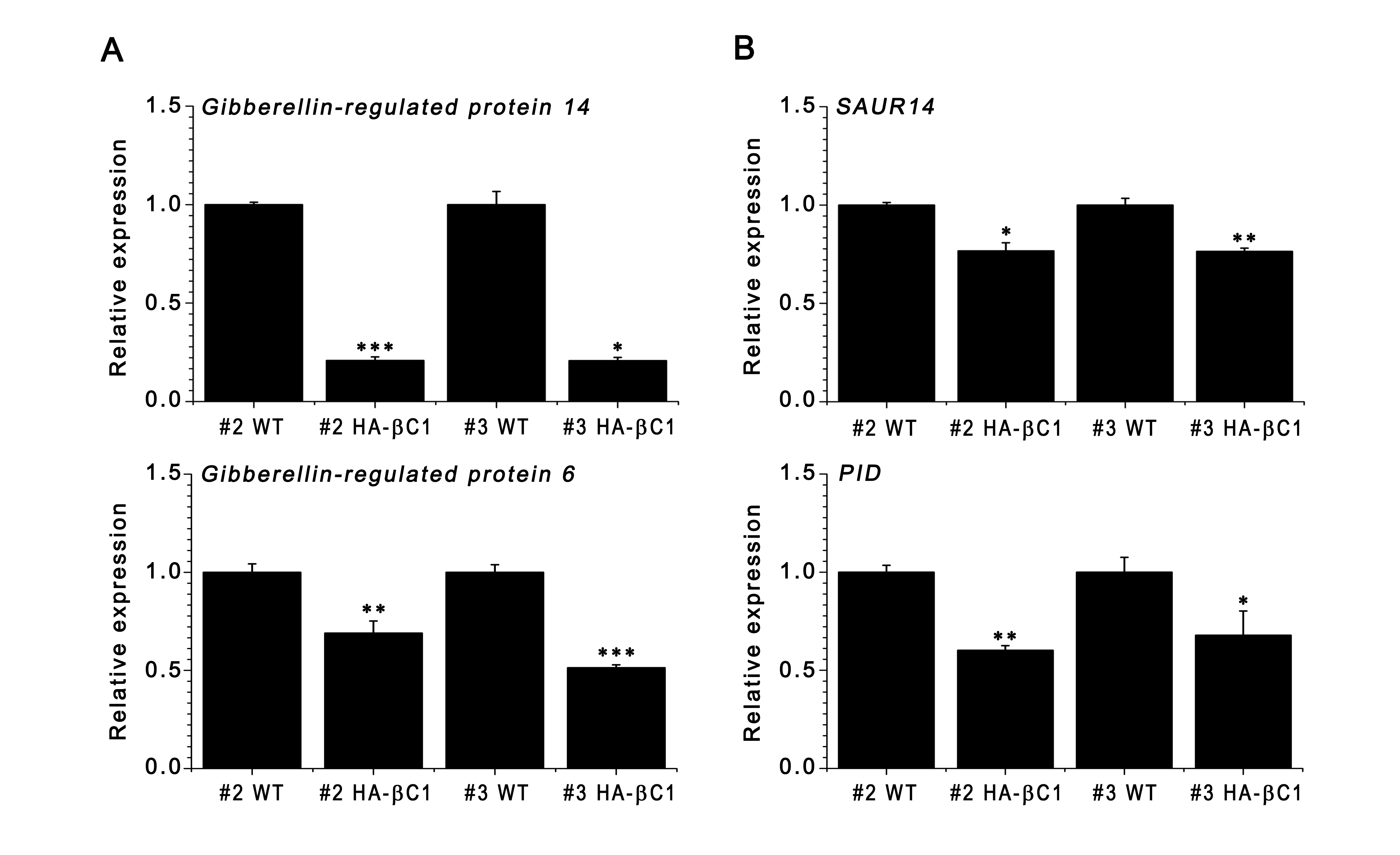

Supplement: S13 Fig — (A) Relative expression level of marker genes of gibberellins response in HA-βC1 transgenic (#2 HA-βC1 and #3 HA-βC1) and wild-type N. benthamiana (#2 Control and #3 Control) seedlings determined by quantitative real-time PCR. #2 HA-βC1 and #2 WT were presented on same plates, while #3 HA-βC1 and #3 WT were presented on same plates. HA-βC1-expressing lines are compared with their corresponding control. (B) Relative expression level of marker genes of gibberellins response in HA-βC1 transgenic and wild-type N. benthamiana (Control) seedlings determined by quantitative real-time PCR. Actin was used as the internal reference. Bars represent SEM. The raw data were analysed by two-sample t-test to show the significance level at 0.05 (*), 0.01 (**) and 0.001 (***). These experiments were repeated at least twice. (TIF) [file ppat.1005668.s013.tif]

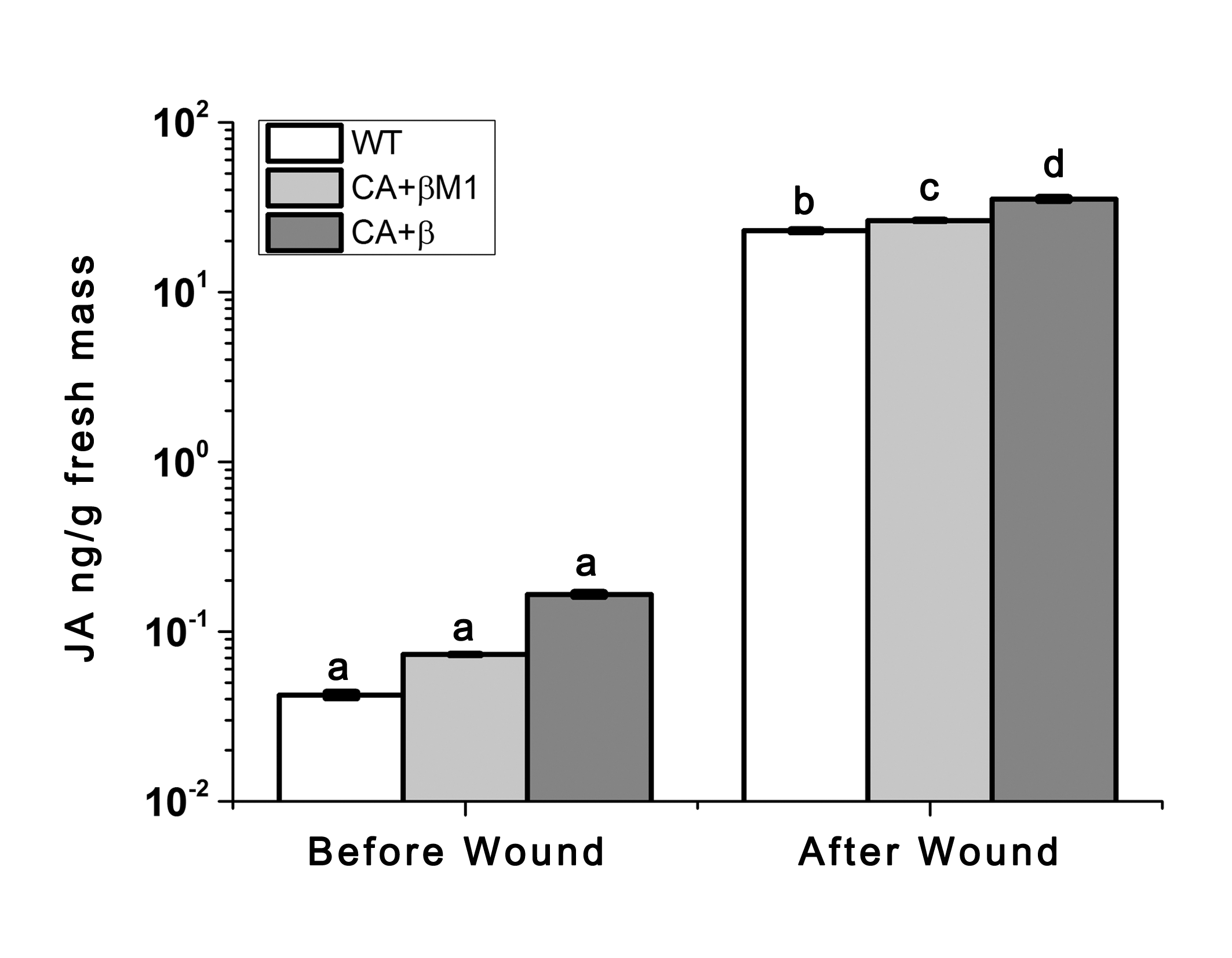

Supplement: S14 Fig — JA levels in healthy N.benthamiana plants or plants infected by CA+β and CA+βM1. Different letters indicate significant differences (ANOVA, P < 0.05). (TIF) [file ppat.1005668.s014.tif]

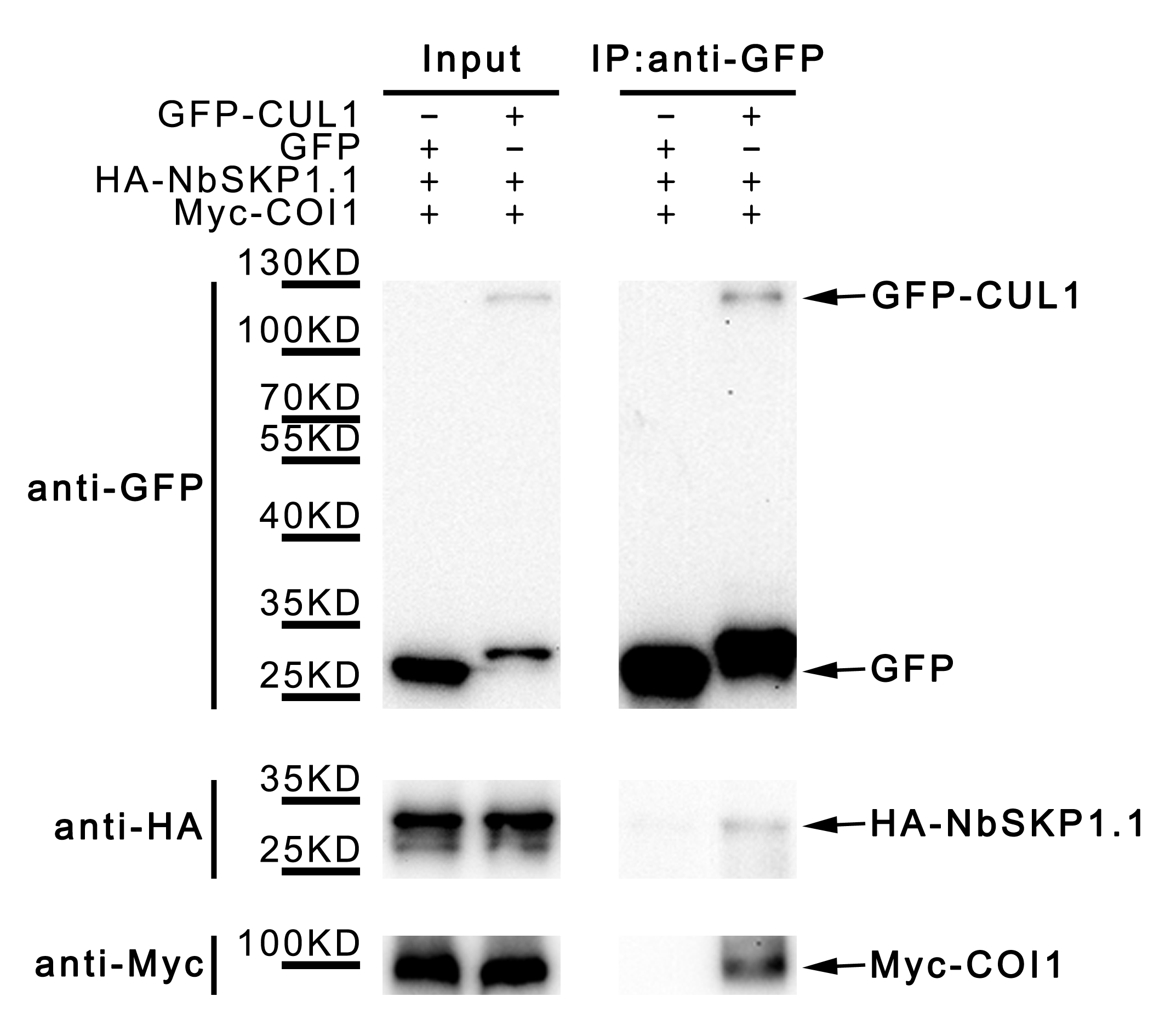

Supplement: S15 Fig — Co-immunoprecipitation (co-IP) assays show that Myc-COI1 interacted with NbSKP1.1 and NbCUL1 in vivo. GFP-CUL1 or GFP (as a negative control) was co-expressed with HA-NbSKP1.1 and Myc-COI1 in N.benthamiana leaves by agroinfiltration. At 48 hpi, leaf lysates were immunoprecipitated (IP) with GFP-Trap agarose, then the immunopercipitates were detected by western blotting using anti-GFP, anti-HA and anti-Myc antibodies. (TIF) [file ppat.1005668.s015.tif]

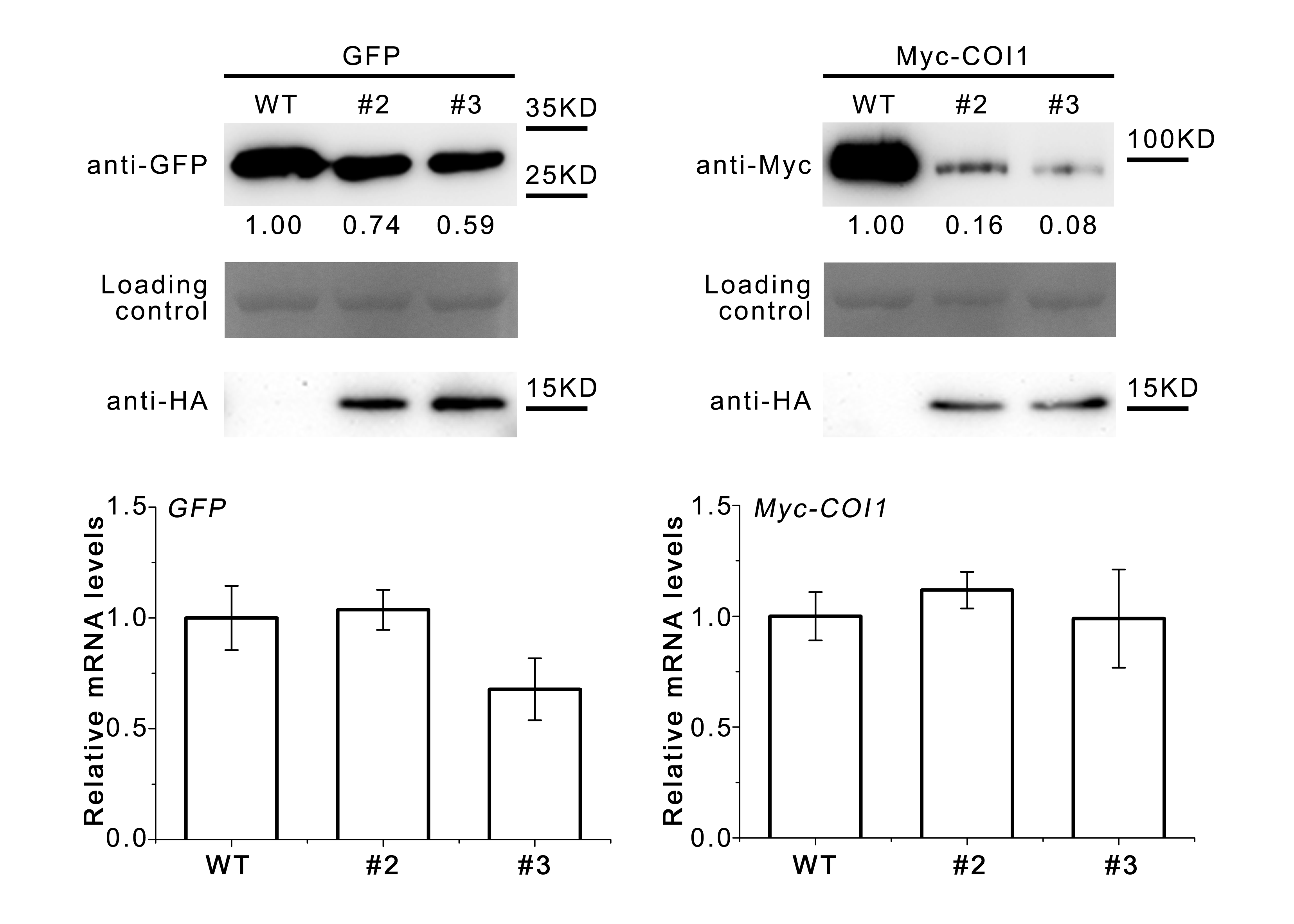

Supplement: S16 Fig — GFP (as the control) or Myc-COI1 was agroinoculated into eight- to nine-week-old wild-type (WT) or HA-βC1 transgenic N. benthamiana plants (#2 and #3). At 48 hpi, leaf lysates were analysed by western blot via anti-Myc or anti-GFP antibody. Intensity was detected through Total Lab TL120. Relative mRNA levels of GFP and Myc-COI1 were quantified via real-time PCR. To exclude influence from endogenous COI, 5’UTR and Myc tag sequences were used to design primers. Actin was used as the internal reference. These experiments were repeated three times. (TIF) [file ppat.1005668.s016.tif]

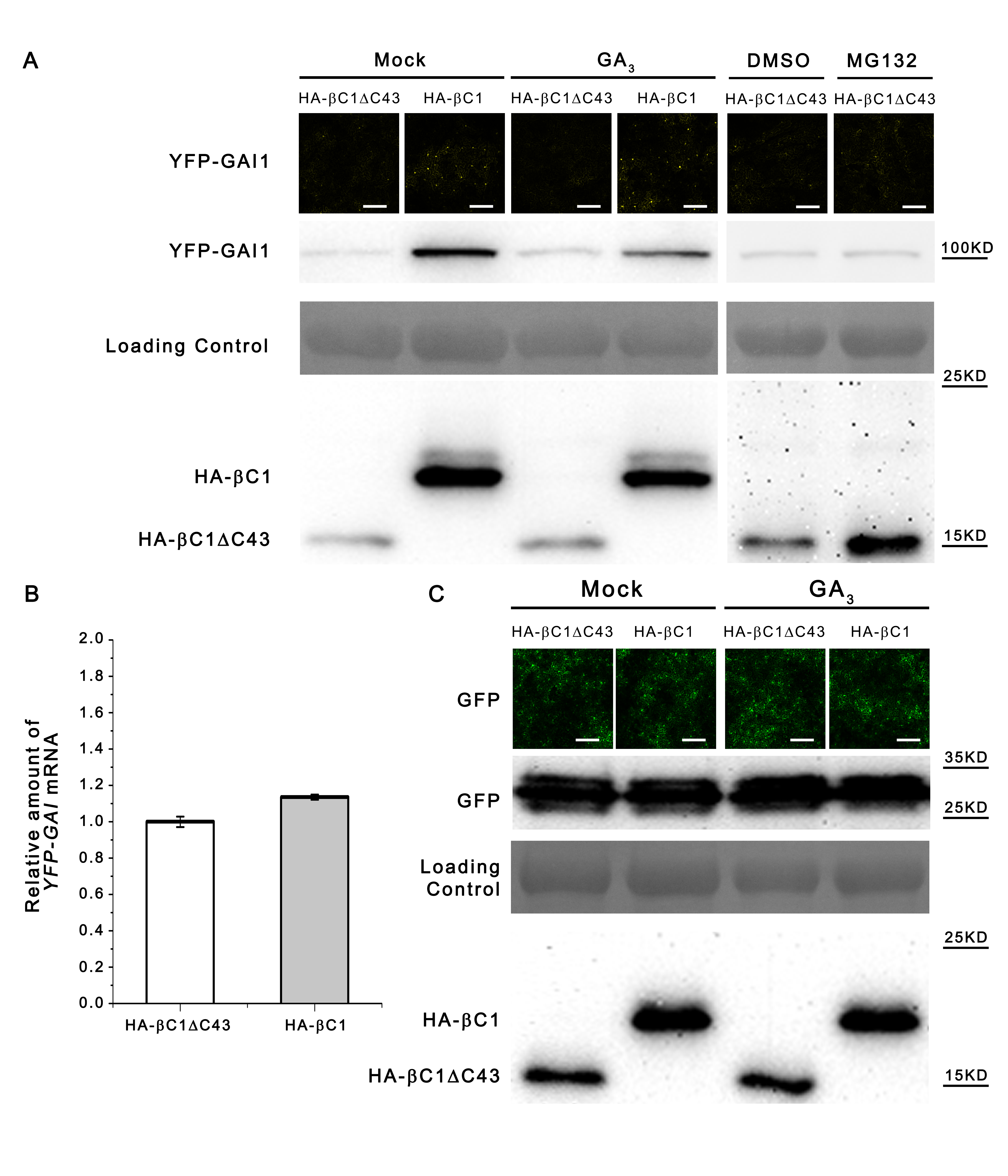

Supplement: S17 Fig — (A) CLCuMuB βC1 attenued degradation of YFP-GAI in vivo. YFP-GAI expression construct was coinfiltrated with constructs expressing HA-βC1ΔC43 or HA-βC1 into seven to eight-week-old N. benthamiana plant leaves. Around 48 hpi, agroinfiltrated leaves were sprayed with 100 μM GA3 or mock solution (ethonal) and visualized via a Zeiss LSM 710 laser scanning microscope. Bar scale represents 200 μm. DMSO and MG132 (50 μM) were applied into plant leaves 12 h before observation. Protein samples were used to do SDS-PAGE and western blot analysis with the anti-GFP antibody, which also recognizes YFP. The PVDF membrane was stained with Ponceaux to visualize the large subunit of ribulose-1,5-bisphosphate as a loading control. (B) Real-time RT-PCR detected the mRNA level of YFP-GAI. Total RNA was extracted from each N. benthamiana leaves and then subjected to quantitative RT-PCR (means±SEM, n = 3) to quantify YFP-GAI mRNA level. Actin was used as the internal reference. (C) CLCuMB βC1 didn’t affect stability of GFP in vivo. Detection of GFP (as an internal control) in N. benthamiana leaves coinfiltrated with the construct expressing GFP together with constructs expressing HA-βC1ΔC43 or HA-βC1 and treated with 100 μM GA3 or mock (ethanol) solution and visualized via a Zeiss LSM 710 laser scanning microscope. Bar scale represents 200 μm. Protein samples were subjected to SDS-PAGE and immunoblot analysis with anti-GFP. The PVDF membrane was stained with Ponceaux to visualize the large subunit of ribulose-1,5-bisphosphate as a loading control. (TIF) [file ppat.1005668.s017.tif]

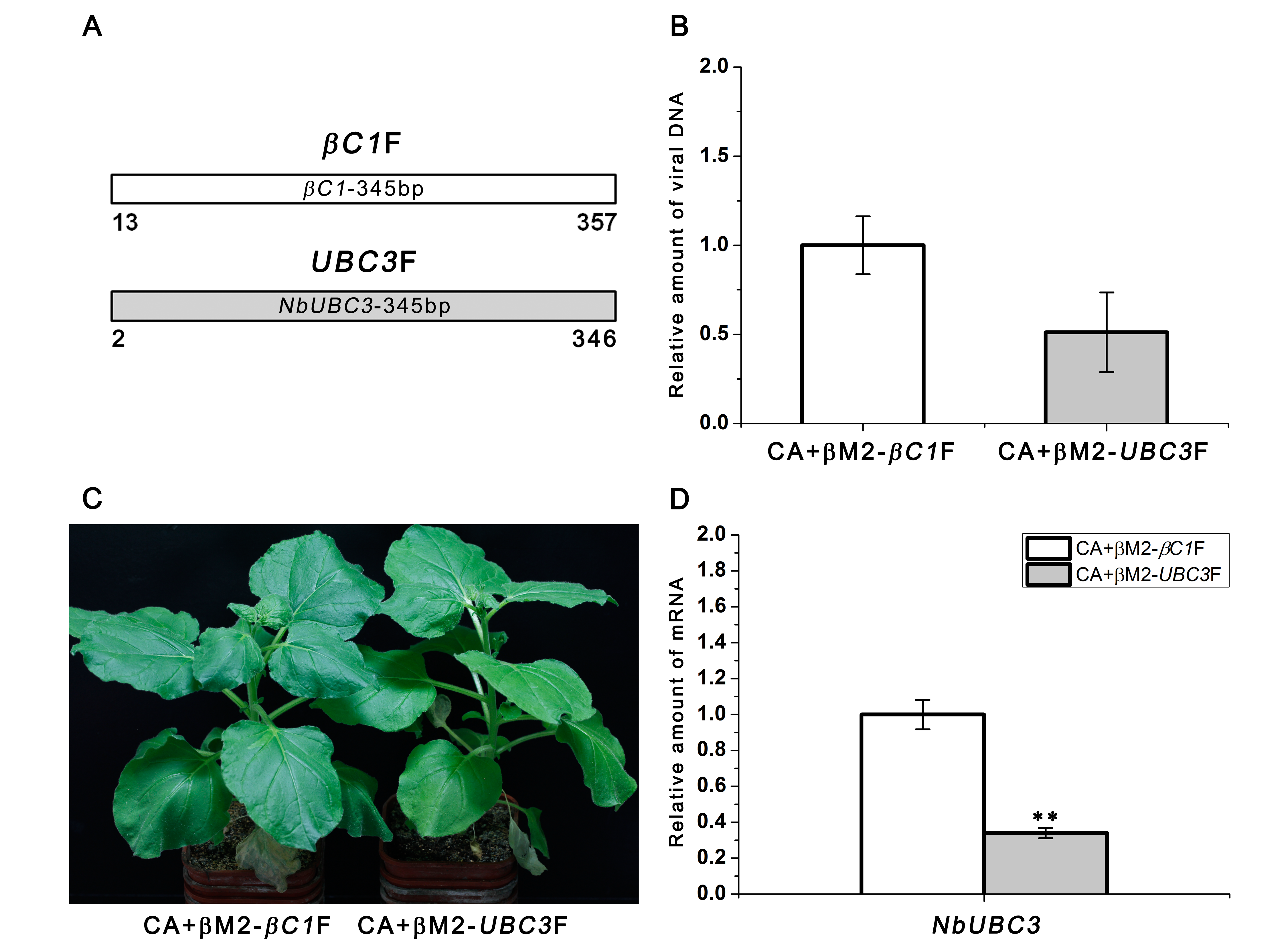

Supplement: S18 Fig — (A) Six- to seven-week-old N. benthamiana plants were agroinoculated with CLCuMuV and βM2-UBC3F which is resulted by introducing a 345-bp fragment of UBC3 into βM2. (B) Silencing of UBC3 led to no enhancement on virus accumulation. 7 plants for each group. At 14 dpi, total DNA was extracted from upper leaves of each plant respectively and subjected to quantitative real-time PCR (means±SEM, n = 7) to quantify viral DNA accumulation. The internal reference method was used to calculate the relative amount of viral DNA. (C) Silencing of UBC3 led to no typical symptom even at 21 dpi. (D) Real-time RT-PCR confirmed silencing of NbSKP1s. Total RNA was extracted from each plant respectively and subjected to quantitative RT-PCR (means±SEM, n = 4) to quantify UBC3 mRNA level. Actin was used as the internal reference. The raw data of (B) and (D) were analysed by two-sample t-test to show the significance level at 0.05 (*). These experiments were repeated at least twice. (TIF) [file ppat.1005668.s018.tif]

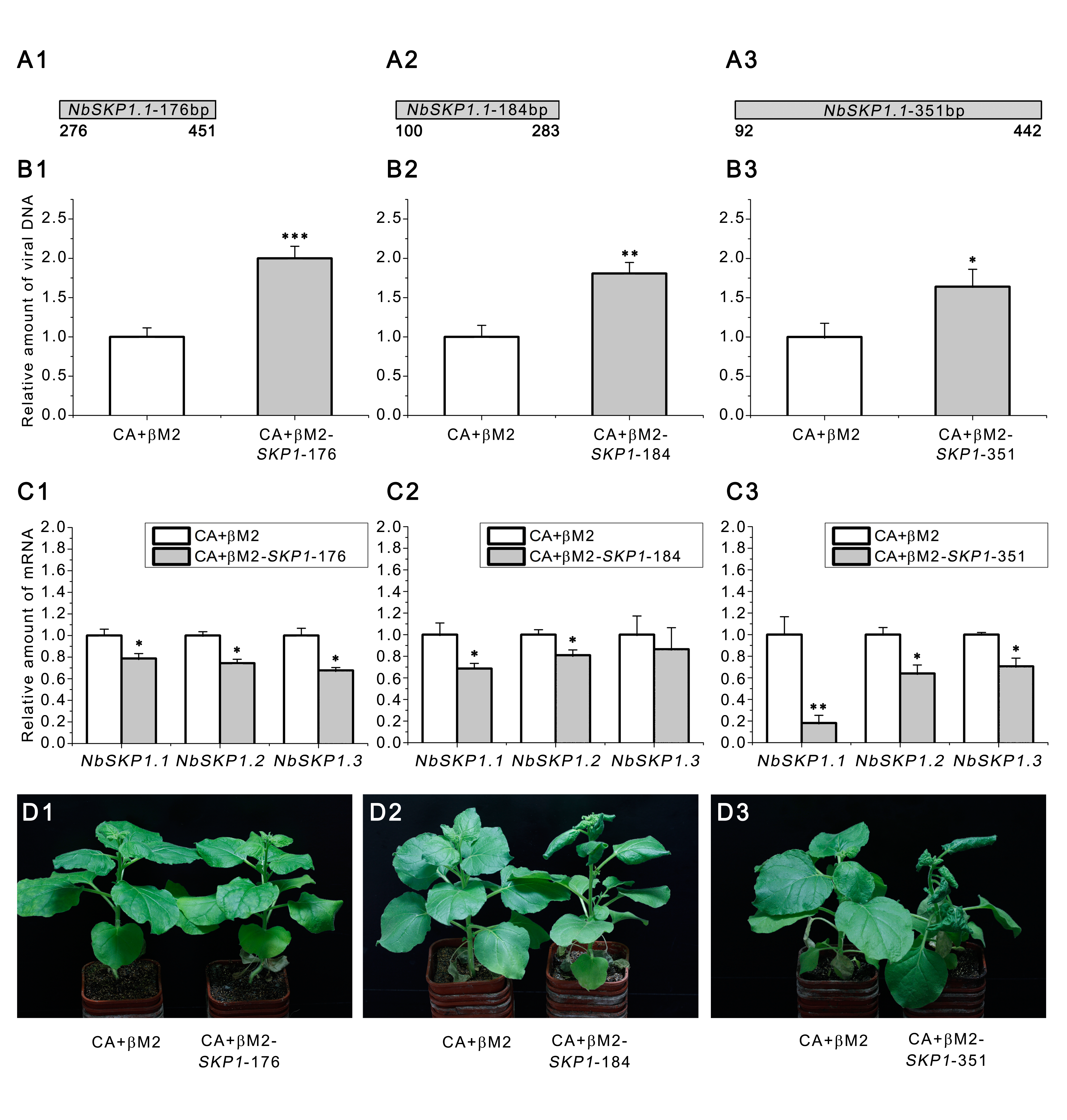

Supplement: S19 Fig — (A1, A2 and A3) Six- to seven-week-old N. benthamiana plants were agroinoculated with CLCuMuV (CA) and βM2-SKP1-176 (A1), βM2-SKP1-184 (A2), βM2-SKP1-351 (A3) or βM2 (as the control). (B1, B2 and B3) Silencing of NbSKP1s enhanced CLCuMuV DNA accumulation. At 14 dpi, total DNA was extracted from each plant respectively and subjected to quantitative real-time PCR (means±SEM, n ≥7) to quantify viral DNA accumulation. EIF4a was used as the internal reference to calculate the relative amount of viral DNA. (C1, C2 and C3) Real-time RT-PCR confirmed silencing of NbSKP1s. Total RNA was extracted from upper leaves of each plant respectively and subjected to quantitative RT-PCR (means±SEM, n = 4) to quantify NbSKP1s mRNA level. EIF4a was used as the internal reference. The raw data of (B1–B3) and (C1–C3) were analysed by two-sample t-test to show the significance level at 0.05 (*), 0.01 (**) and 0.001(***). These experiments were repeated at least twice. (D1, D2 and D3) Symptoms of plants infected with CLCuMuV (CA) and βM2-SKP1-176 (A1), βM2-SKP1-184 (A2) or βM2-SKP1-351 (A3) at 21 dpi. No plants infected with CA+βM2-SKP1-176, about 50% plants infected with CA+βM2-SKP1-184 and all plants infected with CA+βM2-SKP1-351 showed typical symptoms. (TIF) [file ppat.1005668.s019.tif]
