# Supplementary material for: CLCuMuB βC1 Subverts Ubiquitination by Interacting with NbSKP1s to Enhance Geminivirus Infection in Nicotiana benthamiana
Source: PLoS Pathog. 2016 Jun 17;12(6):e1005668. doi: 10.1371/journal.ppat.1005668 (PMC4912122; doi:10.1371/journal.ppat.1005668)
Supplement: S1 Table — (PDF) [file ppat.1005668.s020.pdf]

S1 Table. Primers Used in Vector Construction and PCR Analysis.

Restriction site used for cloning is underlined.

Name	Sequence	Restriction site	Used for
*Hind*III-A-F	CGG<u>AAGCTT</u>AGATTTGCATTTAAATTATG	*Hind*III	pCA
*Xba*I-A-R	GCAAA<u>TCTAGA</u>CAGGATCTTTCAGGAG	*Xba*I	pCA
*Xba*I-A-F	GCTCCTGAAAGATCCTG<u>TCTAGA</u>TTTGC	*Xba*I	pCA
*Kpn*I-A-R	CGG<u>GGTACC</u>AATCAAAGTACAGCACAGG	*Kpn*I	pCA
*Kpn*I-β-F	CGG<u>GGTACC</u>GTATATGCCTGTATATACG	*Kpn*I	pβ
*Hind*III-β-R	CATGTATA<u>AAGCTT</u>TCAAGCCAACATC	*Hind*III	pβ
*Hind*III-β-F	GATGTTGGCTTGA<u>AAGCTT</u>TATACATG	*Hind*III	pβ
*Sac*I-β-R	CGG<u>GAGCTC</u>CCAACACATGTAATACATCTCC	*Sac*I	pβ
βM1-F	TGAAATAAGCAGATTGAACGAGGAGCAGAACAAACACG		pβM1
βM1-R	GTTCTGCTCCTCGTTCAATCTGCTTATTTCATGGAATTGTGTA TTAT		pβM1
*Kpn*I-βM2-F	CGG<u>GGTACC</u>ATGACAAACACAAACCAGCAATGC	*Kpn*I	pβM2
*Xba*I-*Asc*I-βM2-R	CGG<u>TCTAGA</u><u>GGCGCGCC</u>TGCTTATTTCATGGAATTGTG	*Xba*I	pβM2

*Xba*I-βM2-F	CGGTCTAGAATGACAAACACAAACCAGCAATGC	*Xba*I	pβM2
*Sac*I-βM2-R	CGGGAGCTCTGCTTATTTCATGGAATTGTG	*Sac*I	pβM2
*Asc*I-*SKP1*-176-F	TCTGGCGCGCCCCTCTTTGATCTCATCTTGGCTGCC	*Asc*I	pβM2-*SKP1*F1;<br>pβM2-*SKP1*-176
βC1ΔC43-F	ATGACGAGGAGCAGAACAAACACG		pHA-βC1ΔC43;<br>pPVX-βC1ΔC43
βC1ΔC43-R	TTAGTTGTATGCGAATAGGAAATTCGCAATGAT		pHA-βC1ΔC43;<br>pPVX-βC1ΔC43
BamHI-*βC1ΔC43*-F	TTAGGATCCACGAGGAGCAGAACAAACACG	*BamHI*	pGST-βC1ΔC43
*Xho*I-*βC1ΔC43*-R	TTACTCGAGTTAGTTGTATGCGAATAGGAAATTCGCAATGAT	*Xho*I	pGST-βC1ΔC43
SKP1-176-*βC1*-R	ATGATGTTCCCTCATTCTCCCTCCTAACCTCCTCTTCTT		pβM2-*SKP1*F1
SKP1-176-*βC1*-F	GGAGGGAGAATGAGGGAACATCATTGCGAATTTCCTATTC		pβM2-*SKP1*F1
*Xba*I-*βC1*F-R	TTATCTAGATTAAACGGTGAACTTCTTATTGAATACGTATG	*Xba*I	pβM2-*SKP1*F1;<br>pβM2-*SKP1*F2;<br>pβM2-*CUL1*F1;<br>pβM2-*βC1*F
*Asc*I -*βC1*F-F	ATCTGGCGCGCCAGAACAAACACGCAGGGAGTCAG	*Asc*I	pβM2-*βC1*F
*Asc*I-*SKP1*-184-F	TCTGGCGCGCCAGCATATGATTGAAGATGATTGCGCC	*Asc*I	pβM2-*SKP1*F2;<br>pβM2-*SKP1*-184

SKP1-184-*βC1*-R	AATTCGCAATGACAAAGAGGGTGGCCTGGTCAAC		pβM2-*SKP1*F2
SKP1-184-*βC1*-F	CCACCCTCTTTGTCATTGCGAATTTCCTATTCGCATACA		pβM2-*SKP1*F2
*Asc*I-*SKP1*-345-F	TCTGGCGCGCCATTGAAGATGATTGCGCCGACAC	*Asc*I	pβM2-*SKP1*F3
*Xba*I-*SKP1*-345- R	TTATCTAGACCTCCTAACCTCCTCTTCTTCCTCT	*Xba*I	pβM2-*SKP1*F3
*Asc*I-*CUL1*-345- F	TCTGGCGCGCCTGGTCTACCAAGAGTTGAATGGCAAAG	*Asc*I	pβM2-*CUL1*F1;<br>pβM2-*CUL1*F2
*Xba*I-*CUL1*-345-R	TTATCTAGACATGTTGTACTTTCTCTAGCAACTTTGTC	*Xba*I	pβM2-*CUL1*F1
*Asc*I-*CUL1*-268- F	TCTGGCGCGCCTGGTCTACCAAGAGTTGAATGGCAAAG	*Asc*I	pβM2-*CUL1*F2
CUL1-268-*βC1*-R	TTTCAAGTACAAAGGCACTCCTCGGCTTTCAGC		pβM2-*CUL1*F2
CUL1-268-*βC1*-F	CGAGGAGTGCCTTTGTACTTGAAAACCCAGAGATATTGGG		pβM2-*CUL1*F2
*Xba*I-*PDS*-F	TTATCTAGAGGGCCCTGACGAGCTTTCGATGCAG	*Xba*I	pβM2-*PDS*
*Asc*I-*PDS* -R	TCTGGCGCGCCATGGACATTTATCACAGGAACTCCCAC	*Asc*I	pβM2-*PDS*
*Asc*I-*UBC3*-345-F	TCTGGCGCGCCTGGCGTCGAAGCGTATATTGAAA	*Asc*I	pβM2-*UBC3*
*Xba*I-*UBC3*-345-R	TTATCTAGACTGGATTTGGATCTGTCAACAGAGAAC	*Xba*I	pβM2-*UBC3*
*Xba*I-*2mβ*-*SKP1*-345-F	TTATCTAGAATTGAAGATGATTGCGCCGACAC	*Xba*I	p2mβ-*SKP1*F3
*BamH*I-*2mβ*-*SKP1*-345<br>-R	TTAGGATCCCCTCCTAACCTCCTCTTCTTCCTCT	*BamH*I	p2mβ-*SKP1*F3
*Xba*I-*2mβ*-*GFP*-345-F	TTATCTAGAGGAGAAGAACTTTTCACTGGAGTTGTC	*Xba*I	p2mβ-*GFP*F
*BamH*I-*2mβ*-*GFP*-345-	TTAGGATCCGGTGTCTCCCTCAAACTTGACTTCAG	*BamH*I	p2mβ-*GFP*F

R			
q*eIF4a*-F	GCTTTGGTCTTGGCACCTACTC		Real-time PCR
q*eIF4a*-R	TGCTCGCATGACCTTTTCAA		Real-time PCR
qCLCuMuV *V1*-F	ACAACAGGCATGGACAAACA		Real-time PCR
qCLCuMuV *V1*-R	CCAATACGATGGGTCAAACC		Real-time PCR
q*NbSKP1.1*-F	GAATGCTTGGGCATTTGAGT		Real-time PCR
q*NbSKP1.1*-R	CAGCAAAAGGAGGTTCAAGC		Real-time PCR
q*NbSKP1.2*-F	CCTGGGCCTTTGAGTGAATC		Real-time PCR
q*NbSKP1.2*-R	ACAAACAGCACGGCATATCT		Real-time PCR
q*NbSKP1.3*-F	AGAAGTCAGGAGGGAGAACG		Real-time PCR
q*NbSKP1.3*-R	AGAGCACAAGAAACCATGCA		Real-time PCR
q*NbSKP1L1*-F	GTGTGGAATGGGACGAATCG		Real-time PCR
q*NbSKP1L1*-R	GGCGGAATAACTACGAATCCAG		Real-time PCR
q*NbCUL1*-F	AGCCAAGCACCAAAACGATT		Real-time PCR
q*NbCUL1*-R	ACCGCCTATCCTTGTCAACA		Real-time PCR
q*Actin*-F	TGCCATTCTCCGTCTTGACT		Real-time PCR
q*Actin*-R	TGCAGTCTCGAGTTCCTGTT		Real-time PCR
q*Defensin-like protein 1*-F	TGCCTTACCAAACCACCATG		Real-time PCR
q*Defensin-like protein*	CCTCAGCTAAAGTTTCAGCTCC		Real-time PCR

-R			
q*Defensin-like protein 2*-F	GCCACTGTGTTACTTCTGGC		Real-time PCR
q*Defensin-like protein 2*-R	CAGTCACCACCGGAAAATCC		Real-time PCR
q*Pathogen like protein* -F	GCTTTCTCACAAGACTCGCG		Real-time PCR
q*Pathogen like protein* -R	ACGTTTATCACTTCCCACGC		Real-time PCR
q*Gibberellin-regulated protein 14*-F	AGCCAGGCAATTCGTGTTTT		Real-time PCR
q*Gibberellin-regulated protein 14*-R	TCCACATGCCCGTATACACA		Real-time PCR
q*Gibberellin-regulated protein 6*-F	GTATGGACCAGGGAGCTTGA		Real-time PCR
q*Gibberellin-regulated protein 6*-R	GGTCCTCCTTCCTTGGTCTT		Real-time PCR
q*SAUR14*-F	GGCTATCCGTGTTCCTCGTA		Real-time PCR
q*SAUR14*-R	GAGCAGGGAATTGTGACACC		Real-time PCR
q*PID*-F	GCAGCAGAGGTTTTGGTAGC		Real-time PCR

q*PID*-R	ATGCAGAAAAATGGTGGAGG		Real-time PCR
q*YFP-GAI*-F	GAGCTACCAGTCCGCCCT		Real-time PCR
q*YFP-GAI*-R	CTTGTACAGCTCGTCCATGC		Real-time PCR
q*GFP*-F	AGAGGGTGAAGGTGATGCAA		Real-time PCR
q*GFP*-R	TTCCCGTCGTCCTTGAAGAA		Real-time PCR
q*MYC-COI1*-F	AGGATCCCATCGATTTAAAGCT		Real-time PCR
q*MYC-COI1*-R	TTGCTCCATGGTGAGGTCG		Real-time PCR
CLCuMuV *βC1* CDS-F	ATGACGAGGAGCAGAACAAACA		pBD-βC1;<br>pGST-βC1;<br>pHA-βC1-nYFP;<br>pHA-βC1;<br>pPVX-βC1
CLCuMuV *βC1*<br>CDS-R	TTAAACGGTGAACTTCTT		pBD-βC1;<br>pGST-βC1;;<br>pHA-βC1;<br>pPVX-βC1
CLCuMuV *βC1* CDS<br>(without stop code)-R	AACGGTGAACTTCTTATTG		pHA-βC1-nYFP
NbSKP1.1 CDS-F	ATGTCGTCCTCTAAGATGATCGTATTGAAG		pAD-NbSKP1.1;

			pAD-N98;<br>pHA-NbSKP1.1;<br>pHis-HA-NbSKP1.1;<br>pHA-cYFP-NbSKP1.1<br>pnYFP-NbSKP1.1
NbSKP1.1 CDS-R	TCACTCAAATGCCCAAGCATTCTCCCTCCT		pAD-NbSKP1.1;<br>pAD-C57;<br>pHA-NbSKP1.1;<br>pHis-HA-NbSKP1.1;<br>pHA-cYFP-NbSKP1.1<br>pnYFP-NbSKP1.1
NbSKP1.1 C57-F	ATGGCTGCCAACTACTTGAACATCAAGAG		pAD-C57
NbSKP1.1 N98-R	TCACAAGATGAGATCAAAGAGGGTGGCC		pAD-N98
NbSKP1.2 CDS-F	ATGTCTACTTCAAAGATGATTGTGTTGAAGAGT		pAD-NbSKP1.2
NbSKP1.2 CDS-R	TCACTCAAAGGCCCAGGCATTCT		pAD-NbSKP1.2
NbSKP1.3 CDS-F	ATGAAGATGATCGTGCTAAGGAGTTCC		pAD-NbSKP1.3

NbSKP1.3 CDS-R	TCACTCGAAGGCCCAGGCGT		pAD-NbSKP1.3
NbSKP1L1 CDS-F	ATGGCTGCCTCCTCATCAGCAT		pAD-NbSKP1L1; pHA-NbSKP1L1; pHis-HA-NbSKP1 L1;
*NbSKP1L1*CDS-R	CTAATAAGTCGATTCGTCCCATTCCACAC		pAD-NbSKP1L1; pHA-NbSKP1L1; pHis-HA-NbSKP1 L1;
NbCUL1 CDS-F	ATGAATCAGCGTACCACAATCGATTTAGAC		pGFP-NbCUL1 pcYFP-NbCUL1
NbCUL1 CDS-R	TCATGCCAAGTATTTGAACAAGTTCGGG		pGFP-NbCUL1 pcYFP-NbCUL1
GAI CDS-F	ATGAAGAGAGATCGTGATAGAGAAAAATCC		pYFP-GAI
GAI CDS-R	CTACAACCCGGCATCGCCG		pYFP-GAI
